# Investigation of relation between cyber hygiene and news veracity judgements

**DOI:** 10.3389/fpubh.2026.1962217

**Published:** 2026-10-26

**Authors:** Sabina Baraković, Nejra Pezo, Jasmina Baraković Husić

**Affiliations:** 1 Faculty of Traffic and Communications, University of Sarajevo, Sarajevo, Bosnia and Herzegovina; 2 Faculty of Electrical Engineering, University of Sarajevo, Sarajevo, Bosnia and Herzegovina

**Keywords:** cyber hygiene, cyber security, fake news, investigation, judgement, relation, veracity

## Abstract

**Introduction:**

Cyber hygiene is often assumed to enhance individuals' ability to evaluate the veracity of online information, with potential implications for decision-making, social trust, and resilience to misinformation. However, empirical evidence regarding the relationship between cyber hygiene and news-veracity judgment remains limited.

**Methods:**

This exploratory cross-sectional study examined associations between cyber hygiene and news-veracity judgment among 306 participants. Cyber hygiene was assessed through measures of awareness, behavior, and knowledge. News-veracity judgment was operationalized through participants' evaluations of six curated news stimuli (four true and two false items).

**Results:**

No consistent relationship was observed between cyber hygiene awareness, behavior, or knowledge and participants' news veracity judgments in the present sample. Although some individual cyber-hygiene knowledge items showed statistically significant associations with specific news stimuli, these effects were not consistent across items and did not indicate a stable overall relationship. However, gender differences emerged, with women displaying greater distrust and skepticism toward news content, which does not imply the increased accuracy in news veracity judgement.

**Discussion:**

The findings provide no evidence of a stable relationship between the measured dimensions of cyber hygiene and news-veracity judgment in this sample. These results suggest that cyber hygiene, as assessed in the present study, may not directly translate into improved evaluation of news credibility. By reporting largely null findings, the study contributes to refining theoretical assumptions regarding misinformation resilience and highlights the need for future research examining the interaction of cyber hygiene, psychosocial factors, and response tendencies in news-veracity judgment.

## 1 Introduction

Cyber hygiene has emerged as a critical dimension of digital security in response to increasingly sophisticated cyber threats and the expanding digital presence of individuals and organizations (1). Traditionally associated with practices such as multi-factor authentication, secure browsing, regular software updates, and phishing awareness (2), cyber hygiene is rooted in the cybersecurity domain. However, recent research suggests that its relevance extends beyond technical protection, intersecting with broader issues of digital literacy and societal resilience (3, 4). In particular, the potential link between cyber hygiene and the news veracity judgement warrants closer theoretical and empirical examination.

Fake news detection has typically been studied within the frameworks of media literacy, digital literacy, and information literacy (5–9). These literatures emphasize skills such as critical evaluation of sources, contextual interpretation, and recognition of misinformation patterns. By contrast, cyber hygiene emphasizes preventive security behaviors (10, 11). The conceptual bridge between these domains lies in the cultivation of cautious, informed, and proactive digital practices (9, 10). For example, phishing awareness requires users to scrutinize suspicious content, a skill that may transfer to evaluating the credibility of online news (12). Similarly, secure browsing and source verification foster habits of skepticism and validation that overlaps with competencies central to misinformation detection (13). Thus, while cyber hygiene and fake news detection originate from distinct traditions, their convergence reflects a broader paradigm of responsible digital citizenship.

Both cyber hygiene and fake news influence individual wellbeing, decision-making, and social trust [(14, 22)], i.e., they impact quality of life (QoL). Strong cyber hygiene can enhance user confidence and reduce anxiety, while exposure to fake news has been shown to erode trust and impair judgment, particularly during crises such as the coronavirus disease 2019 (COVID-19) pandemic (15). Existing literature highlights the prevalence and impact of fake news globally, yet much of this evidence remains descriptive and dispersed across technical, psychological, and sociological domains (16–18). While reports suggest that misinformation circulates faster than verified content and undermines confidence in institutions (19, 20), there is limited synthesis of how preventive digital practices might mitigate these outcomes. This gap points to the need for research that explicitly connects cybersecurity-oriented behaviors with misinformation detection capacities.

Bosnia and Herzegovina offers a particularly relevant empirical context. As a society characterized by complex political structures, diverse media ecosystems, and ongoing challenges of post-conflict reconciliation, the country is highly exposed to both cyber threats and misinformation campaigns. Studying this context not only addresses a local need but also provides insights into how cyber hygiene and fake news detection interact in environments where trust in institutions and media is fragile. To the authors' knowledge, this is the first comprehensive study to examine the relationship between cyber hygiene and news veracity judgement in Bosnia and Herzegovina.

Accordingly, the primary aim of this exploratory study is to investigate whether individuals with stronger cyber hygiene awareness, behaviors, and knowledge demonstrate greater accuracy in detecting fake news. By clarifying the conceptual link between cybersecurity practices and misinformation resilience, this research contributes to both theoretical debates and practical strategies for enhancing digital wellbeing.

This study offers valuable insights applicable across the entire cyber hygiene landscape. Various stakeholders can benefit in distinct ways, including: (i) research institutions engaged in investigations of the relationship between fake news and cyber hygiene, (ii) policy makers and government agencies involved in shaping guidelines and strategies for effective cyber hygiene practices to counter fake news, (iii) public and private organizations responsible for promoting and implementing cyber hygiene measures including internal trainings, (iv) media and journalism outlets promoting media literacy and providing accurate reporting, (v) social media platforms responsible for detecting and removing fake news, (vi) civil society and non-governmental organizations (NGOs) interested in promoting informed citizenship and protecting vulnerable communities.

The remainder of this paper is organized as follows. Section 2 provides background information on the topic. Section 3 outlines the research methodology employed in conducting the study. Section 4 presents an analysis of the findings. Section 5 offers a discussion that highlights the study's interpretation of findings. Finally, Section 6 concludes the paper, acknowledges its limitations, and provides relevant recommendations for future work.

## 2 Theoretical background

### 2.1 Cyber hygiene

Cyber hygiene can be defined as the set of habitual practices, behaviors, and technical measures that individuals and organizations adopt to maintain the security, integrity, and resilience of their digital environments (2, 11). It is framed as a preventive discipline, like public health hygiene, but applied to cyber space (21). As such, it can be measured using multi-dimensional framework called cyber hygiene inventory (CHI), which contains items and categorizes them into five dimensions, i.e., storage and device hygiene, transmission and browsing hygiene, Facebook and social media, authentication and credential hygiene, and e-mail and messaging hygiene (22).

Cyber hygiene requires maintaining secure devices, safe internet and social media practices, strong authentication habits, and effective email awareness, all of which together strengthen users' ability to avoid cyberattacks. It is widely understood depending on three user focused outcomes: knowledge, awareness, and behavioral practices (21, 23, 24). Knowledge and awareness reflect what users know and understand, while behavioral practices capture how they apply this understanding in real situations (25). Strong cyber hygiene emerges when informed users recognize what actions are required and carry them out when needed.

Recent trends in cyber hygiene outcomes point to increased user knowledge and awareness. Users are becoming more aware of cyber threats, with many recognizing the importance of practices like password management and phishing awareness (10). Institutions are implementing programs to enhance cybersecurity education, particularly among students, highlighting the need for comprehensive training to improve understanding of cyber hygiene (26). However, despite increased knowledge, many users still exhibit poor cyber hygiene behaviors, such as inadequate device security and lack of two-factor authentication (26). Furthermore, organizations often struggle to enforce cyber hygiene policies, leading to a disconnect between knowledge and practical application (27).

While there is a positive trend in awareness and knowledge regarding cyber hygiene, the challenge remains in translating this understanding into consistent, effective behaviors. In other words, while knowledge and awareness are crucial, they do not always translate into effective behavior change. Users may still fall victim to fake news despite understanding its risks, indicating a need for more robust strategies to foster genuine behavioral shifts in response to fake news (28).

### 2.2 Fake news

To date no universally accepted definition of fake news has been established, even within the field of journalism. However, establishing a precise and comprehensive definition is essential for building a robust foundation for its analysis. Several concepts often overlap with fake news, including cherry-picking (29), clickbait (30), satirical news (31), rumors (32), deceptive news (33), false information (34), disinformation and misinformation (35). A widely cited definition describes fake news as “news articles that are intentionally and verifiably false, and that could mislead readers” (36), emphasizing intentionality and verifiability as core elements.

While definitional clarity and technological detection methods are important, the ability of individuals to recognize and resist fake news is equally critical. Research shows that psychological factors such as confirmation bias and emotional reactivity, combined with limited digital skills and lack of reflective reasoning, make users more vulnerable to misinformation (37). Thus, individual-level determinants (i.e., analytic thinking, reflective reasoning, motivated cognition, and source evaluation) constitute the cognitive and epistemic pathways through which detection ability is shaped. Strengthening these skills through media literacy programs and critical thinking initiatives enables individuals to distinguish reliable information from falsehoods (38, 39). These educational approaches complement technological solutions, creating a more comprehensive strategy for combating fake news.

From technological perspective, fake news can be analyzed in terms of knowledge, propagation, style and source (40). Detection strategies manual fact-checking, either through experts' verification or crowd-sourced evaluation (41), as well as automated methods based on machine learning and deep learning (40, 42). However, these methods are most effective when paired with individual-level competencies that foster epistemic vigilance and behavioral practices of information hygiene.

The spread of fake news has implications for QoL. Exposure to misinformation can erode trust, increase anxiety, and reduce willingness to adopt beneficial technologies, thereby affecting both personal wellbeing and workplace productivity (43). Therefore, combating fake news is not only a matter of cybersecurity but also of safeguarding psychological health and social stability (16, 44). Effective interventions must therefore combine technological tools, educational initiatives, and individual-level skill development to mitigate the broader harms of fake news (45).

Ultimately, the persistence and rapid evolution of fake news present ongoing challenges, including its role as a cybersecurity threat (46). Addressing these challenges requires continuous adaptation and collaboration across disciplines. Effective interventions must combine technological tools, educational initiatives, and individual-level skill development to mitigate the broader harms of fake news, thereby protecting both information integrity and quality of life.

### 2.3 Link between cyber hygiene and fake news

The relationship between cyber hygiene and fake news is based on the idea that secure digital behavior may support individuals' ability to evaluate online information. Users with greater cybersecurity knowledge may be better able to recognize and avoid threats, while literacy-related skills can improve news-veracity judgment (10, 47). However, general awareness of fake news alone may have limited influence on resistance to false information. Knowledge of specific manipulation techniques and the ability to critically evaluate information appear to be more important for identifying misleading content (28, 48). Cyber hygiene may therefore be connected to news-veracity judgment through cognitive and behavioral mechanisms such as analytic thinking, reflective reasoning, source evaluation, and verification of information (49, 50). These practices may also encourage skepticism and fact-checking, potentially reducing the influence of prior beliefs when evaluating information (51, 52).

The relationship may also operate in the opposite direction. Exposure to fake news can influence individuals' behavior and attitudes toward technology, as observed during the COVID-19 pandemic, while understanding the psychological factors underlying trust in false information may support improvements in cybersecurity behavior (28, 53). Information-hygiene practices, digital literacy, and technical approaches for assessing the trustworthiness of online information have consequently been proposed as ways of addressing misinformation (8, 54, 55). Research has also examined social-media sharing behaviors and technical indicators for identifying fake news, as well as the effects of training on news-veracity judgment, with age and education potentially influencing performance (56–58).

These issues are also relevant in organizational environments, where fake news can contribute to financial and reputational risks, while effective cyber hygiene can support trust and a more productive working environment (59, 60). At the same time, organizational culture, financial constraints, limited awareness, and knowledge gaps may hinder effective cyber hygiene practices (61, 62). Thus, existing literature suggests a multifaceted and potentially bidirectional relationship between cyber hygiene and fake news, involving technical, cognitive, behavioral, and organizational factors. Nevertheless, the existence of these conceptual links does not establish that individuals with stronger cyber hygiene actually make more accurate news-veracity judgments. Direct empirical evidence examining whether cyber hygiene awareness, behavior, and knowledge are associated with judgments of individual true and false news stimuli remains limited. This gap motivates the present exploratory study.

However, to the authors' best knowledge, there are no empirical studies that prove the positive connection between cyber hygiene and news veracity judgement. In other words, the existing research studies intuitively assume that if one has quality cyber hygiene habits, he or she will be able to detect fake news more frequently and this will have a positive impact on their lives. Therefore, in this paper, we aim to test this assumed positive relationship and confirm or deny its existence.

## 3 Research methodology

This section describes the research methodology used to collect data in this study. Subsection 3.1. gives research questions resulting from the background literature review from previous section. Survey design and procedure are described in subsection 3.2., while more details regarding survey participants are given in subsections 3.3.

### 3.1 Research questions

In order achieve the abovementioned aims and goals, we have formed the following research questions:

RQ1: is there a relation between gender and the judgement of news veracity?

RQ2: is there a relation between age and the judgement of news veracity?

RQ3: is there a relation between cyber hygiene awareness and the judgement of news veracity?

RQ4: is there a relation between cyber hygiene behavior and the judgement of news veracity?

RQ5: is there a relation between cyber hygiene knowledge and the judgement of news veracity?

### 3.2 Study design and procedure

To test the relationship, we have used a survey questionnaire, which comprises of two parts: cyber hygiene part and fake news part. Cyber hygiene part of the questionnaire used in this survey study, its development process, and check of its construct, criterion, and content validity has already been described in (23). These 30 questions are given in Table 1 in the results section since they also contain results of the collected answers. This part covers cyber hygiene awareness (Q7, Q20, Q27–Q30), behavior (Q3–Q6, Q9–Q10, Q12–Q14), and knowledge questions (Q11, Q15–Q19, Q21–Q26). Because the cyber hygiene instrument comprises heterogeneous item-level indicators reflecting distinct practices, awareness components, and knowledge domains, the questionnaire was not treated as a reflective latent scale and internal consistency coefficients were not considered the primary form of measurement evaluation. Instead, evidence regarding content validity and instrument development has been reported previously (23).

Table 1Summary of results of the cyber hygiene part of the questionnaire.

**Table:** 

Cyber hygiene survey questions and results			
Q1	How old are you?	Mean	
		**23.11**	
Q2	What gender are you?	Male	Female
		**49.7%**	**50.3%**

**Table:** 

Q3	The devices I use to access the Internet are:				
	Desktop/laptop computer	Tablet	Smartphone	Smart wearables	Other
	**90.5%**	**12.7%**	**96.1%**	**8.8%**	**5.9%**

**Table:** 

Q4	The computer I use can be accessed by						
	Me, exclusively	Me and my parents	Me and my siblings	Me and other extended family members	Me and my partner	Me and friends	All
	**61%**	**15.4%**	**24.5%**	**3.9%**	**7.8%**	**2.9%**	**4.6%**
Q5	The smartphone/device I use can be accessed by						
	Me, exclusively	Me and my parents	Me and my siblings	Me and other extended family members	Me and partner	Me and friends	All
	**87.6%**	**4.2%**	**3.3%**	**0.3%**	**5.9%**	**2.6%**	**1.3%**

**Table:** 

Q6	The applications I have on my smartphone/device are used for							
	Internet search	Social networks	Shopping	Financial management	Games	Security management	Learning	Other
	**91.8%**	**97.4%**	**59.2%**	**49.3%**	**61.4%**	**18.6%**	**65.4%**	**15%**

**Table:** 

Q7	On which devices that you use are security solutions enabled (antivirus, antimalware, etc.)?				
	At all	On the computer	On a smartphone/device	Not at all	Other
	**40.8%**	**39.2%**	**8.2%**	**9.8%**	**2%**

**Table:** 

Q8	If you don't use security solutions on your smartphone/device or computer, why don't you do it?							
	I hate it	There is no need	Because it takes up too much storage space	Too many options from which I don't know which one to choose	Too expensive	It takes too much time	Slows down the device	Other
	**33.2%**	**28.3%**	**6.5%**	**14.1%**	**10.3%**	**4.3%**	**13%**	**32.1%**

**Table:** 

Q9	I create my passwords…				
	By combining letters, numbers and symbols and 8 characters	With at least 8 characters	From private information (date of birth, name, etc.)	All of the above	None of the above
	**55.9%**	**4.9%**	**10.5%**	**20.3%**	**8.5%**

**Table:** 

Q10	How do you manage your passwords?					
	I store my passwords on a computer or write them down on paper	I use the same passwords for most occasions	I store the password on a browser (Mozilla, Chrome, etc.)	I do not hide my passwords – everyone knows them	I remember them	I store my passwords with the help of password manager
	**36.6%**	**28.8%**	**14.7%**	**0.7%**	**50.3%**	**28.4%**

**Table:** 

Q11	Which of the following passwords is the most secure?					
	Most123	PoG#&78	lokum78	123456	Admin	I am not sure
	**1.3%**	**91.5%**	**2%**	**0.3**	**0%**	**4.9%**

**Table:** 

Q12	What personal information do I share through the devices I use to access the Internet? This includes social networks (Instagram, Facebook, etc.), e-mail (gmail.com, etc.), communication applications (Viber, WhatsApp, etc.), web search, etc.					
	Name and surname	Registration number	Images	Video	Documents	Opinions, feelings, statements, etc.
	**92.2%**	**2%**	**74.5%**	**44.4%**	**16.7%**	**32.7%**

**Table:** 

	Home address	Relationship status	Phone number	Location	Bank card number	Plans	Other
	**9.2%**	**13.1%**	**32%**	**19.6%**	**4.2%**	**4.6%**	**5.9%**

**Table:** 

Q13	Do you use public Wi-Fi networks?			
	I don't use public Wi-Fi networks	I use password-protected public Wi-Fi networks	I use public Wi-Fi networks that are familiar to me	I use public Wi-Fi networks that are available and free
	**26.5%**	**31%**	**29.4%**	**43.5%**

**Table:** 

Q14	I use Wi-Fi network…				
	For social networks (including Viber, WhatsApp, etc.)	For e-mail communication	For online shopping	For banking transactions	For other applications
	**95.1%**	**56.9%**	**44.8%**	**34.6%**	**44.4%**

**Table:** 

Q15	If a public Wi-Fi network (café, station, etc.) requires an access password, is it safe to use it for sensitive activities such as online or mobile banking?		
	Yes	No	Not sure
	**11.8%**	**48.7%**	**39.5%**

**Table:** 

Q16	All Wi-Fi traffic is encrypted by default on all wireless routers.		
	No	Yes	Not sure
	**15.7%**	**20.9%**	**63.4%**

**Table:** 

Q17	What does https:// at the beginning of the URL mean compared to http://?					
	That the website has high definition	That the entered information is encrypted	That it is the latest available version of the website	That the website cannot be accessed from certain computers	None of the above	Not sure
	**13.1%**	**29.4%**	**3.3%**	**1.6%**	**5.9%**	**46.7%**

**Table:** 

Q18	“Private search” is a feature of many Internet browsers that allows users to access the website without any information (such as search history) that the browser can store. Do Internet Service Providers (ISPs) see their subscribers' online activities when they use the “private search” option?		
	Yes	No	Not sure
	**52.3%**	**5.2%**	**42.5%**

**Table:** 

Q19	All e-mails are encrypted by default		
	Correct	Incorrect	Not sure
	**27.8%**	**10.5%**	**61.8%**

**Table:** 

Q20	Do you check that the files you download or the devices you use are infected with viruses before you use them?				
	Yes, both	Files only	Devices only	No, because there is no need	No, because it takes too much time
	**51.3%**	**14.1%**	**4.2%**	**8.2%**	**22.2%**

**Table:** 

Q21	Which of the following is an example of phishing?				
	When someone is sent an e-mail that contains a malicious link disguised as looking like an e-mail from a celebrity	Creating a fake website that looks approximately identical to the actual website in order to deceive the user and enter their information	When a text message is sent to someone containing a malicious link disguised as looking like a notification that a person has won a contest or lottery	All of the above	Not sure
	**12.7%**	**15%**	**8.5%**	**28.1%**	**35.6%**

**Table:** 

Q22	Some websites and online services use a security process called two-factor authentication. Which of the following images is an example of two-factor authentication?					
	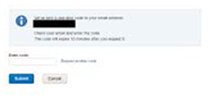	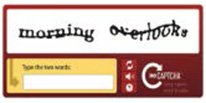	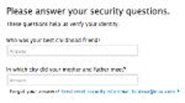	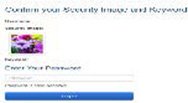	None of the above	Not sure
	**44.1%**	**17.3%**	**32.7%**	**17.6%**	**3.3%**	**22.2%**

**Table:** 

Q23	A group of computers that are networked and used by hackers to steal information is called:				
	Botnet	Rootkit	DDoS	Operating system	Not sure
	**27.1%**	**8.5%**	**14.7%**	**2%**	**47.7%**

**Table:** 

Q24	Criminals enter the computer and encrypt the user's personal files and data. The user is unable to access this data unless he pays criminals to decrypt the files. This is called:					
	Botnet	Ransomware	DDoS	Spam	None of the above	Not sure
	**6.2%**	**38.9%**	**4.9%**	**2.6%**	**2.9%**	**44.4%**

**Table:** 

Q25	Turning off the Global Positioning System (GPS) feature on your smartphone makes it impossible to track your phone's location.		
	Correct	Incorrect	Not sure
	**37.3%**	**41.5%**	**21.2%**

**Table:** 

Q26	What types of security risks are reduced by using virtual private networks (VPNs)?					
	Phishing attacks	Monitoring by website operators	Use of insecure Wi-Fi networks	Deanonymization by network providers	Writing keys	Not sure
	**9.2%**	**23.9%**	**15.7%**	**9.5%**	**3.6%**	**38.2%**

**Table:** 

Q27	I back up my data on devices…				
	Once a month	Once in 3 months	Once in 6 months	Once a year	Never
	**27.8%**	**12.7%**	**7.8%**	**17%**	**34.6%**

**Table:** 

Q28	Do you regularly update software on the smart devices you use?			
	Yes	No, because it is not necessary	No, because it takes too much time	No, because it doesn't matter to me
	**76.5%**	**5.2%**	**6.5%**	**11.8%**

**Table:** 

Q29	The links I use most often to download software and applications on my computer are:		
	• Checked and safe	• Unverified and insecure	It depends
	**63.4%**	**5.6%**	**31%**

**Table:** 

Q30	The links I use most often to download software and applications on my smartphone/device are:		
	• Checked and safe	• Unverified and insecure	It depends
	**68.3%**	**5.9%**	**25.8%**

In order to examine the relationship between cyber hygiene awareness, behavior, and knowledge and participants' judgments of news veracity, the questionnaire was amended with an additional set of fake-news-related questions (10 questions in Table 2). The primary outcome was operationalized as participants' veracity judgments of six individual news items (i.e., whether each item was judged to be true or false), rather than as a fully validated measure of general fake-news detection accuracy. The most important part of the fake news related questions are the ones where we offered 6 news for participants to assess whether they are true or false. First four news were true, while the remaining two were false.

Table 2Summary of results of the fake news part of the questionnaire.

**Table:** 

Fake news survey questions and results					
Q31	How often do you come across the news which you suspect are fake?				
	Daily	Weekly	Monthly	Rarely	Never
	**53.3%**	**23.5%**	**8.8%**	**11.4%**	**2.9%**

**Table:** 

Q32	On which platforms you mostly notice fake news?				
	Social networks	Online portals and blogs	Television	Print media	I do not notice any
	**85.9%**	**62.1%**	**21.9%**	**14.7%**	**3.3%**

**Table:** 

Q33	How do you check the accuracy of the information?			
	I am searching for other sources of information	I am using fact-checking sites	I ask family or friends	I do not check
	**67.6%**	**7.5%**	**9.5%**	**15.4%**

**Table:** 

Q34	Do you think that fake news has a significant impact on society?			
	Yes, very large	Yes, but not crucial	No, they have small influence	No, they do not impact
	**63.4%**	**27.1%**	**6.2%**	**3.3%**

**Table:** 

Q35	What are the main reasons for fake news dissemination in your opinion?				
	Manipulation of public opinion	Gaining profit from clicks	Political interests	Lack of media regulation	Journalism flaws
	**79.7%**	**68.35**	**61.85**	**34.3%**	**15.4%**

**Table:** 

Q36	Have you ever shared news that turned out to be fake?		
	Yes, consciously	Yes, unconsciously	No, never
	**8.2%**	**47.7%**	**44.1%**

**Table:** 

Q37	Which measures would be the most effective in fighting fake news in your opinion?				
	Better education regarding media literacy	More strict regulation and laws	Higher responsibility on social networks	Stronger engagement of fact-checking organizations	Fake news detection tools
	**69.3%**	**61.1%**	**56.9%**	**33%**	**47.7%**

**Table:** 

Q38	Have you ever used any fact-checking tools	Yes	No
		**3.9%**	**96.1%**

**Table:** 

Q40a	Is the following news true of fake? February 2024 is globally the hottest in the history of measurement.	True (Correctly recognized)	Fake (Marked incorrectly)
		**62.7%**	**37.3%**

**Table:** 

Q40b	Is the following news true of fake? NASA launched the Jupiter moon research mission.	True (Correctly recognized)	Fake (Marked incorrectly)
		**45.8%**	**54.2%**

**Table:** 

Q40c	Is the following news true of fake? The lake in India became pink over the night.	True (Correctly recognized)	Fake **(Marked incorrectly)**
		**50.3%**	**49.7%**

**Table:** 

Q40d	Is the following news true of fake? Dinosaurs found in Argentina.	True (Correctly recognized)	Fake **(Marked incorrectly)**
		**22.6%**	**77.4%**

**Table:** 

Q40e	Is the following news true of fake? Oncologist warns on cancer at COVID19 vaccinated.	True (Marked incorrectly)	Fake (Correctly recognized)
		**30.1%**	**69.9%**

**Table:** 

Q40f	Is the following news true of fake? A dramatic twist: scientist declared the sudden end of climate crisis.	True (Marked incorrectly)	Fake (Correctly recognized)
		**18.9%**	**81.1%**

Q40a (TRUE): copernicus Climate Change Service. Copernicus: February 2024 was globally the warmest on record, global sea surface temperatures at record high.

https://climate.copernicus.eu/copernicus-february-2024-was-globally-warmest-record-global-sea-surface-temperatures-record-high

Q40b (TRUE): NASA. Europa Clipper: exploring Jupiter's Ocean Moon – Mission Overview.

https://science.nasa.gov/missions/europa-clipper/europa-clipper-resources/europa-clipper-exploring-jupiters-ocean-moon-mission-overview/

Q40c (TRUE): NASA Earth Observatory. Lonar Lake Tries on a Rosy Color.

https://science.nasa.gov/earth/earth-observatory/lonar-lake-tries-on-a-rosy-color-146859/

Q40d (TRUE): National Geographic. New Carnivorous Dinosaur Species Found in Argentina.

https://news.nationalgeographic.org/new-carnivorous-dinosaur-species-found-in-argentina/

Q40e (FAKE): Public Health Collaborative. Oncologist Falsely Claims COVID-19 Vaccines Are Accelerating Cancers.

https://publichealthcollaborative.org/alerts/oncologist-falsely-claims-covid-19-vaccines-are-accelerating-cancers/

Q40f (FAKE): Raskrinkavanje. Scientists Have Not Declared the End of the Climate Crisis.

https://raskrinkavanje.ba/en/analysis/scientists-have-not-declared-the-end-of-the-climate-crisis.

The six news items were selected using a structured but pragmatic stimulus-selection procedure designed to provide variation in topical domain, presentation format, plausibility, and veracity while maintaining comparability in length, presentation style, and informational complexity. The items were drawn from publicly circulating online content and included both true and false examples. Four items were classified as true and two as false on the basis of the information available from the sources used to establish their veracity. The inclusion of both veracity categories was intended to reduce the likelihood that participants could obtain correct responses simply by consistently selecting one response category. Also, the six news stimuli were presented in the same order to all participants. Randomization of stimulus order was not implemented because the questionnaire was administered using a fixed survey structure.

At the same time, the unequal distribution of true and false items (four vs. two) represents an important limitation of the measurement approach. It means that responses may partly reflect a general tendency to accept or reject news content rather than balanced detection accuracy. Therefore, the results are interpreted primarily in terms of participants' veracity-judgment patterns and item-level associations, rather than as a psychometrically validated measure of general fake-news detection accuracy. The six items were used as exploratory stimuli for examining whether cyber hygiene characteristics were associated with responses to news-veracity judgments.

All items were reviewed by the authors to establish their factual status prior to inclusion. In other words, no formal expert-panel validation or inter-rater reliability assessment was conducted. No formal plausibility rating or pilot-testing procedure was conducted. Four items reflected verified true reports and two reflected demonstrably false claims. Because this module was developed specifically for the present exploratory study, it should not be considered a psychometrically validated fake-news detection scale. In addition, the fake-news module was exploratory and did not undergo formal psychometric validation prior to deployment. Consequently, evidence regarding reliability, construct validity, or measurement invariance is not available and should be established in future studies. The procedure of the study is given in Figure 1.

**Figure 1:**
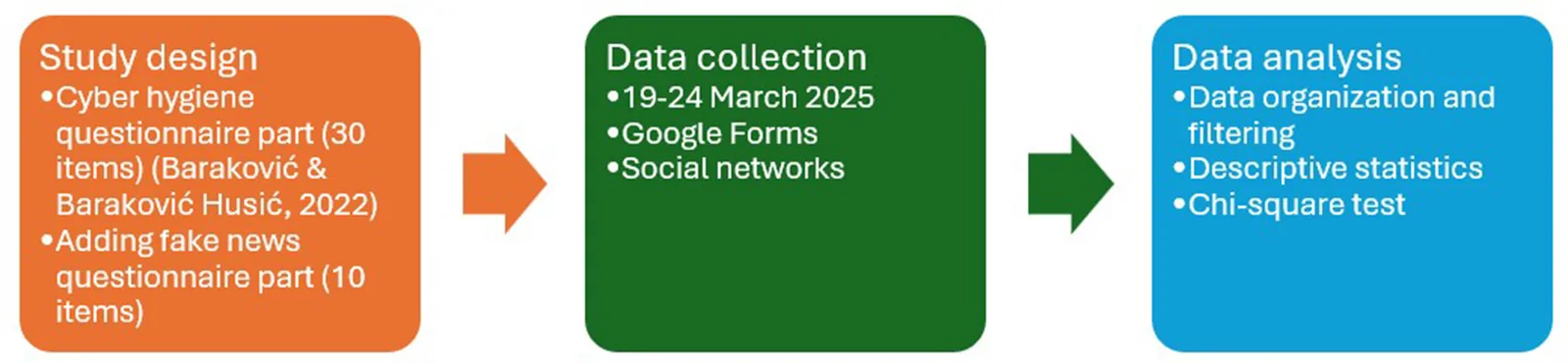
Study procedure.

After designing the questionnaire (Phase 1), it was distributed in Google Forms in period from March 19th, 2025 to March 24th, 2025 and via various social networks thereby easing the fast and simple reach to broader scope of people (Phase 2). The questionnaire was administered in Bosnian, which is the native language of the target population.

The participants were informed before completing the questionnaire that participation was voluntary, that responses would be collected anonymously, and that all information would be treated confidentially. Participation was entirely optional and respondents could discontinue the survey at any time before submission. Completion and submission of the anonymous questionnaire were regarded as informed consent to participate. Ethical approval was not required in accordance with local legislation and institutional requirements because the study involved an anonymous online survey and did not collect personally identifiable or sensitive information. All participants provided responses to the news-veracity items; therefore, no pairwise or listwise exclusion due to missing data was required for the fake-news analyses. In other words, no missing cyber hygiene responses were identified and therefore, no imputation procedures or exclusions were required.

Data collected by the survey was entered into Microsoft Excel and subsequently analyzed using IBM SPSS Statistics (86) (Phase 3). Descriptive statistics and Pearson's Chi-square tests of independence were used to examine associations between categorical variables. The Chi-square analyses compared participants' responses to individual cyber hygiene questions with their judgments of the veracity of each of the six news items. Statistical significance was initially assessed at *p* ≤ 0.05.

For each Chi-square analysis, degrees of freedom and Cramer's *V* were considered together with the *p*-value in order to assess both statistical significance and the magnitude of the association. The assumptions underlying the Chi-square test, including the distribution of expected cell frequencies, were also considered when interpreting the results. In general, effect sizes were quantified using Cramér's *V* and interpreted according to conventional thresholds (approximately 0.10 = small, 0.30 = medium, and 0.50 = large). Given the categorical nature of the analyses and the use of χ^2^ tests of independence, effect sizes are reported together with χ^2^ statistics, degrees of freedom, and *p*-values. As a robustness analysis, *p*-values were additionally evaluated using the Benjamini-Hochberg false-discovery-rate (FDR) procedure within each family of related comparisons (gender, age, awareness, behavior, and knowledge analyses). Adjusted significance was assessed at *q* = 0.05.

Most contingency tables met the minimum expected frequency assumptions for Pearson's Chi-square test. However, for age-related analyses involving five age categories, some expected frequencies fell below the conventional threshold because of the small number of participants in the oldest age group (50+ years; *n* = 10). Consequently, age-related findings were interpreted cautiously and supplemented with robustness analyses using collapsed age categories.

Given the large number of item-level comparisons performed in this exploratory study, the nominal *p* ≤ 0.05 threshold was not interpreted as providing confirmatory evidence for individual associations. Instead, statistically significant item-level results were considered hypothesis-generating and were interpreted in the context of the overall pattern of findings, effect sizes, and consistency across the six news stimuli. No causal interpretation was made because the study used a cross-sectional observational design.

The study is primarily exploratory and focuses on identifying associations between variables rather than developing predictive models. Accordingly, the item-level analyses were retained to provide a detailed examination of the relationships corresponding to the research questions, while avoiding population-level or causal interpretation of isolated statistically significant findings. Item-level analyses were selected because aggregate scores may mask heterogeneous relationships among specific cyber-hygiene components. The separate Chi-square analyses were used as an exploratory screening approach to examine whether specific cyber-hygiene items were associated with judgments of individual news stimuli. They were not intended to provide a full participant-level model of the repeated-measures structure; consequently, the resulting associations should be interpreted as item-specific exploratory evidence rather than as estimates accounting for within-participant dependence. While the present study employed exploratory Chi-square analyses to identify potential associations between categorical variables, future research should consider multivariable approaches such as logistic regression, generalized linear models, or structural equation modeling to examine these relationships within a more comprehensive analytical framework.

### 3.3 Study participants

In total, 306 participants took part in the survey. The data were collected through online distribution via social media platforms, constituting a non-probabilistic convenience sample rather than a probability-based sample. Therefore, the achieved sample size should not be interpreted as establishing population representativeness. In particular, the sample composition and recruitment procedure limit the extent to which the findings can be generalized to the population of Bosnia and Herzegovina. The achieved sample was used for the exploratory examination of associations within the collected dataset. However, the relatively small numbers in the older age categories limit the precision of subgroup comparisons. Accordingly, the findings are not presented as population estimates, and future studies should use formal *a priori* power calculations based on prespecified effect sizes and probability-based sampling designs. This means that no *a priori* power analysis was conducted for this exploratory study. Although no *a priori* power analysis was conducted because the study was exploratory and based on an obtained convenience sample, a sensitivity assessment indicates that the achieved sample size of *N* = 306 did not provide uniformly high power to detect small associations in the item-level Chi-square analyses. At α = 0.05, the approximate power to detect a Cramér's *V* of 0.10 was about 0.42 for one degree of freedom and about 0.25 for four degrees of freedom. For *V* = 0.15, the corresponding power was approximately 0.75 and 0.53, respectively. Thus, the present sample was more capable of detecting moderate or larger associations than very small effects. Accordingly, the non-significant findings should not be interpreted as evidence that small associations are absent.

The gender distribution in the sample was relatively balanced (49.7% male, 50.3% female). However, the age distribution was strongly skewed toward younger participants (range: 16–62 years; *M* = 23.11; SD = 9.04; 0–19: 124, 20–29: 138; 30–39:19; 40–49: 14; 50+: 10), indicating an overrepresentation of younger, digitally active individuals. Consequently, the findings should be interpreted primarily as evidence of associations within the obtained sample rather than as estimates representative of the general population of Bosnia and Herzegovina. The age imbalance also limits the interpretation of age-related findings, as the sample provides substantially greater representation of younger participants than of older age groups. In addition, the age categories (0–19, 20–29, 30–39, 40–49, and 50+) were selected to broadly correspond to commonly used demographic life-stage groupings while retaining sufficient observations per category for exploratory analyses.

The sample is therefore considered appropriate for the exploratory, association-focused purpose of the study, but not for making population-level inferences. Future research should employ larger and more diverse probability-based or stratified samples, with greater representation across age groups and other demographic characteristics, in order to assess whether the observed relationships are consistent across the broader population.

## 4 Results

To answer the posed research questions, we have analyzed the collected data in various ways. Subsection 4.1 contains percentages of answers to cyber hygiene and fake news part of the questionnaire and serves to describe the state of the cyber hygiene of the participants as well as their behavior regarding fake news and the judgement of news veracity. Subsection 4.2 contains analysis of the relation between cyber hygiene awareness and participants' news veracity judgement fake news accurately. Subsequently, subsections 4.3 and 4.4 give the analyses of the relation between cyber hygiene behavior and knowledge and participants' accurate judgement of news veracity. Finally, subsection 4.4 summarizes the results and answers to posed research questions.

### 4.1 The summary of results of the questionnaire

The resulting numbers after the analysis of the collected data in cyber hygiene part of the questionnaire are given in Table 1. The behavior of the participants in terms of cyber hygiene questions is concerning. The participants mostly use desktop/laptop computers and their smartphones to access Internet. In majority, they exclusively are using their computers (61%) and smartphones (87.6%). These devices are mostly used for web browsing, social networks, gaming, shopping, and learning, and less for financial and security management. Enabled security programmes on all devices are present in 40.8% of participants, while 39.2% use them only on computers. They create their passwords by combining letters, numbers and symbols and 8 characters in 55.9% cases, while other offered options were less represented. In terms of management of the passwords, more than half remembers them, while other options such as storing them on computer or writing down on the paper, using the same ones for most of occasions and using password manager are less represented.

According to the survey results, participants share mostly name and surname, images, video, and opinions and feelings, while other options were less represented. When it comes to their behavior in terms of wireless fidelity (Wi-Fi) usage, the participants in 43.5% use networks that are available and free, which is a concerning cyber hygiene behavior. Third of participants use password protected public Wi-Fi networks and ones familiar to them, and don't use Wi-Fi networks. These networks are mostly used for social networks, instant messaging applications, and e-mail communication.

The participants of this survey in 51.3% cases check files and devices they use to see if they are infected, but alarmingly high percentage (22.2%) does not do that since it takes to much time. One third of them backup their data once a month, while one third does not perform backup. The remaining third back up once in 3, 6, or 12 months. Cyber hygiene awareness is good in terms of regularly updating software on smart devices, since 76.5% of participants do it regularly. Good awareness reflects also in almost 2/3 of participants using checked and safe links to download software and applications on computer or smartphone/device.

Cyber hygiene knowledge of survey participants can be marked as medium. Namely, the percentages of correct answers to the knowledge questions, except Q11 (correct password) where we have 91.5% of correct answers, varies. For example, the correct answer to Q15 question is given by only 11.8% of participants, Q16 only 15.7%, or Q19 only 10.5%, which is quite low for Wi-Fi related questions, since many use it daily. On the other hand, on questions Q17 and Q18, which examine participants knowledge regarding hypertext transfer protocol (https) and private search, almost one third to one half of participants gave the correct answer (29.4 and 52.3% respectively). The same situation is with question dealing with the recognition of attacks such as phishing (28.1%), ransomware (38.9%), or botnet (27.1%). Over 40% of participants answered correctly to questions examining their knowledge on two-factor authentication and global positioning system (GPS).

To sum, the level of cyber hygiene behavior of examined survey participants can be assessed as critical, awareness is quite good, and knowledge ranges from low to medium.

Fake news related questionnaire part results are given in Table 2. According to the results of the survey, over half of participants come across fake news daily, and one quarter on a week basis. Mostly fake news is found on social networks and online portals and blogs, while television and print media are less represented. Participants mostly deal with fake news by searching other sources of information (67.6%). Also, the opinion of most of the surveyed participants is that fake news has large influence on the society, and that the main reasons for their production and dissemination are manipulation of public opinion, gaining profit, and political interests. Also, over half of participants think that the effective solution in fighting fake news is better education in media literacy, more strict regulation and laws, higher responsibility on social networks and fake news detection tools (although they have never used one). However, almost half of the participants unconsciously shared fake news.

When asked to evaluate the veracity of the six news items, participants' responses varied substantially across individual stimuli. The first four news items were classified as true, and 62.7%, 45.8, 50.3, and 22.6% of participants correctly identified news 1, news 2, news 3, and news 4, respectively. The remaining two items were classified as false, and 69.9 and 81.1% of participants correctly identified news 5 and news 6 as false. These results indicate substantial variation in participants' veracity judgments across individual stimuli. The higher proportion of “false” judgments for the two false items should not, however, be interpreted as evidence of general fake-news detection accuracy, given the unequal number of true and false stimuli. The pattern may also reflect differences in response tendency or skepticism across items.

Sensitivity (true-positive rate) and specificity (true-negative rate) were first calculated at the participant level based on responses to the four true and two false news items. Participant-level values were then aggregated to obtain sample means and standard deviations. Signal detection theory indices (*d*′ and criterion *c*) were computed from the corresponding hit and false-alarm rates using standard formulas. Because the instrument contained only two false items, individual specificity values could take only three possible values (0, 50, or 100%), which limits measurement precision and produces relatively coarse-grained estimates. Therefore, sensitivity, specificity, balanced accuracy, and signal-detection indices should be interpreted as approximate indicators of performance rather than highly precise measures. Sensitivity, defined as the correct identification of false news items, was 79.2%. Specificity, defined as the correct identification of true news items, was 47.2%. The resulting balanced accuracy was 63.2%. Participants therefore showed substantially greater success in identifying false than true news items. Signal detection analysis further revealed a low discrimination ability (*d*′ = 0.33) and a negative response criterion (*c* = −0.23), indicating a tendency to classify news items as false. These findings suggest that responses reflected not only veracity discrimination but also a skeptical response tendency.

Balanced accuracy was 63.2%, indicating moderate discrimination performance. However, this estimate should be interpreted cautiously because it is derived from an instrument containing four true items and only two false items. The limited number of false items constrains the precision of specificity estimates and affects the stability of balanced accuracy.

When it comes to gender relation, Figure 2 shows that male participants show more believe in truth of the news, while female participants show distrust. In other words, gender was associated with participants' veracity judgments for some individual news items. This is visible from the graph where male participants in 2nd and 3rd case marked true news to be true more than female participants. This trend is also evident in last two examples of fake news that were fake. The difference in approach is also supported by Chi-square test results (news 1, χ^2^(1, *N* = 306) = 0.17, *p* = 0.678, *V* = 0.02 news 2, χ^2^(1, *N* = 306) = 8.12, *p* = 0.004, *V* = 0.16, news 3, χ^2^(1, *N* = 306) = 4.73, *p* = 0.030, *V* = 0.12, news 4, χ^2^(1, *N* = 306) = 0.01, *p* = 0.980, *V* = 0.00, news 5, χ^2^(1, *N* = 306) = 2.22, *p* = 0.136, *V* = 0.09, and news 6, χ^2^(1, *N* = 306) = 5.28, *p* = 0.022, *V* = 0.13; with df = 1). As a robustness analysis, the six gender-related comparisons were additionally evaluated using the Benjamini-Hochberg false-discovery-rate procedure. Following adjustment, only the association between gender and News 2 remained statistically significant, whereas the associations involving News 3 and News 6 were no longer significant. Therefore, the evidence for gender-related differences remained limited and stimulus-specific. The observed pattern indicated a greater tendency among female participants to classify certain items as false, whereas male participants more frequently classified the same items as true. However, these differences should be interpreted as differences in veracity-judgment tendency rather than as evidence that one gender demonstrated greater fake-news detection accuracy. Because the six-item set contained four true and two false stimuli, the observed response pattern cannot establish balanced accuracy or general superiority in detecting fake news. In other words, the answer to RQ1 is: there is a relation between gender news veracity judgement in terms that women show greater distrust. This, however, does not mean that women tend to recognize fake news with greater accuracy. These findings suggest differences in response tendency or skepticism rather than evidence of superior balanced accuracy in fake-news detection.

**Figure 2:**
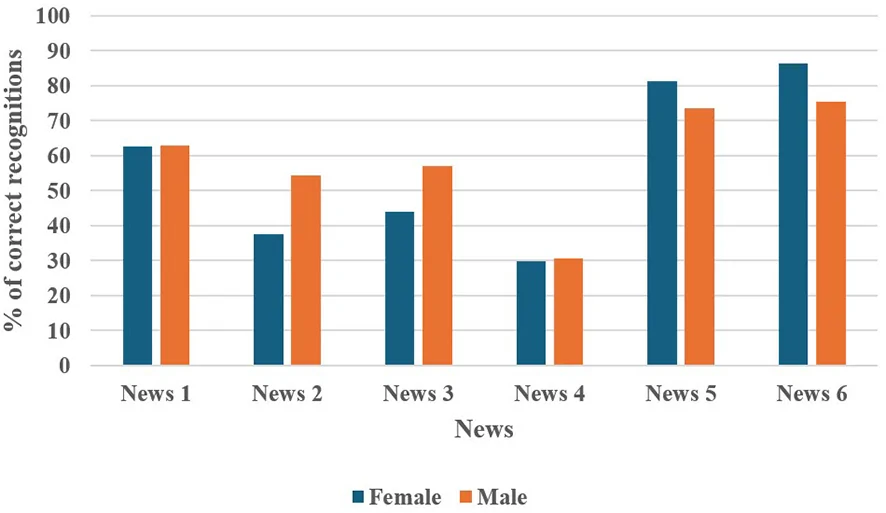
The % of correct recognitions of news veracity in dependence of gender.

As shown in Figure 3, no systematic differences in the percentage of correct answers were observed across age groups in relation to news veracity judgement performance. The graph is supported by Chi-square test results: (news 1, χ^2^(4, *N* = 306) = 4.12, *p* = 0.390, *V* = 0.12, news 2, χ^2^(4, *N* = 306) = 4.20, *p* = 0.380, *V* = 0.12, news 3, χ^2^(4, *N* = 306) = 6.62, *p* = 0.157, *V* = 0.15, news 4, χ^2^(4, *N* = 306) = 8.39, *p* = 0.078, *V* = 0.17, news 5, χ^2^(4, *N* = 306) = 7.62, *p* = 0.107, *V* = 0.16, and news 6, χ^2^(4, *N* = 306) = 7.33, *p* = 0.170, *V* = 0.24; with df = 4). Although some variation can be observed at the item level (e.g., news 4, where correctness rates differed between age groups, and news 6, where certain age groups showed slightly higher correct identification), these patterns were not consistent across all news items. In some cases, different age groups demonstrated higher accuracy depending on the specific item, indicating that performance differences are likely stimulus-dependent rather than age-dependent. False-discovery-rate correction was also applied to the six age-related comparisons. No associations remained statistically significant following adjustment, further supporting the conclusion that no consistent age-related differences were observed.

**Figure 3:**
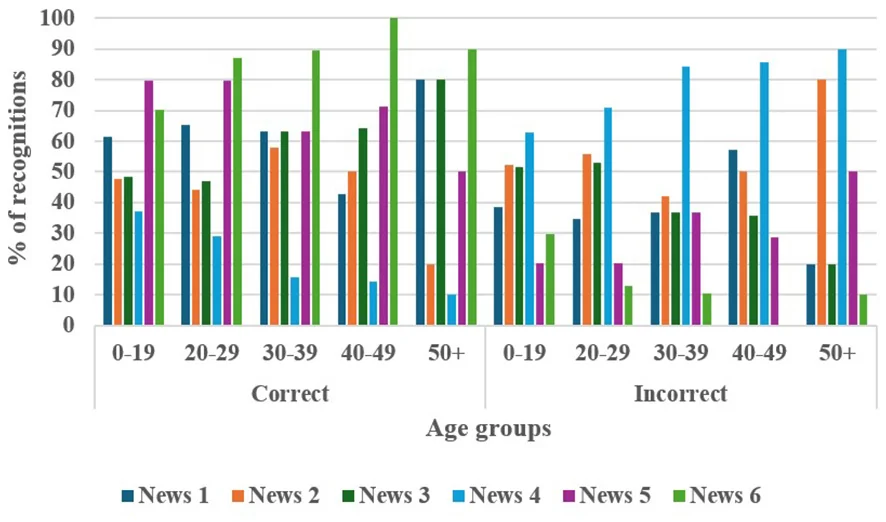
The % of correct recognitions of news veracity in dependence of age group.

Although age-related differences were examined using five age categories (0–19, 20–29, 30–39, 40-49, and 50+ years), the oldest age group contained only 10 participants. Consequently, some expected cell frequencies in the age × item contingency tables fell below the conventional threshold of five. The minimum expected frequency across the six contingency tables was 1.90. Therefore, age-related Chi-square findings should be interpreted with caution.

To assess the robustness of the findings, supplementary analyses were conducted using collapsed age categories (≤29 years vs. ≥30 years). These analyses yielded statistically significant associations for news 3 (χ^2^ = 6.77, *p* = 0.034, *V* = 0.149), news 4 (χ^2^ = 6.69, *p* = 0.035, *V* = 0.148), and news 5 (χ^2^ = 6.39, *p* = 0.041, *V* = 0.145), whereas news 1, news 2, and news 6 remained non-significant. However, all observed effect sizes were small (*V* < 0.20), and no consistent pattern of age-related differences emerged across items. Accordingly, the overall interpretation of the age-related findings remained unchanged.

It is also important to note that the sample shows a strong overrepresentation of younger participants (*M* = 23.11 years), which limits the ability to draw robust conclusions regarding age-related effects. The uneven age distribution, combined with the absence of a consistent pattern across items, suggests that no reliable age-related differences in news veracity judgment can be inferred from this dataset. Therefore, regarding RQ2, the results indicate that age was not a consistent predictor of news veracity judgement performance within the limitations of the present sample structure.

### 4.2 The relation between cyber hygiene awareness and the news veracity judgement

From graphs given in Figures 4, 5, one cannot conclude that cyber hygiene awareness item dealing with enabling security features on different devices has any relation to the news veracity judgement. The percentage of correct answers is the same, from 50 to 60%, with only option (enabling security solutions on other devices) slightly stands out. When analyzed based on each individual news, there is also no significant difference. Namely, first four news are equally recognized as fake by all options, while the remaining two are to a higher extent (80% and above) recognized as true news by participants regardless of their awareness in enabling security features on different devices.

**Figure 4:**
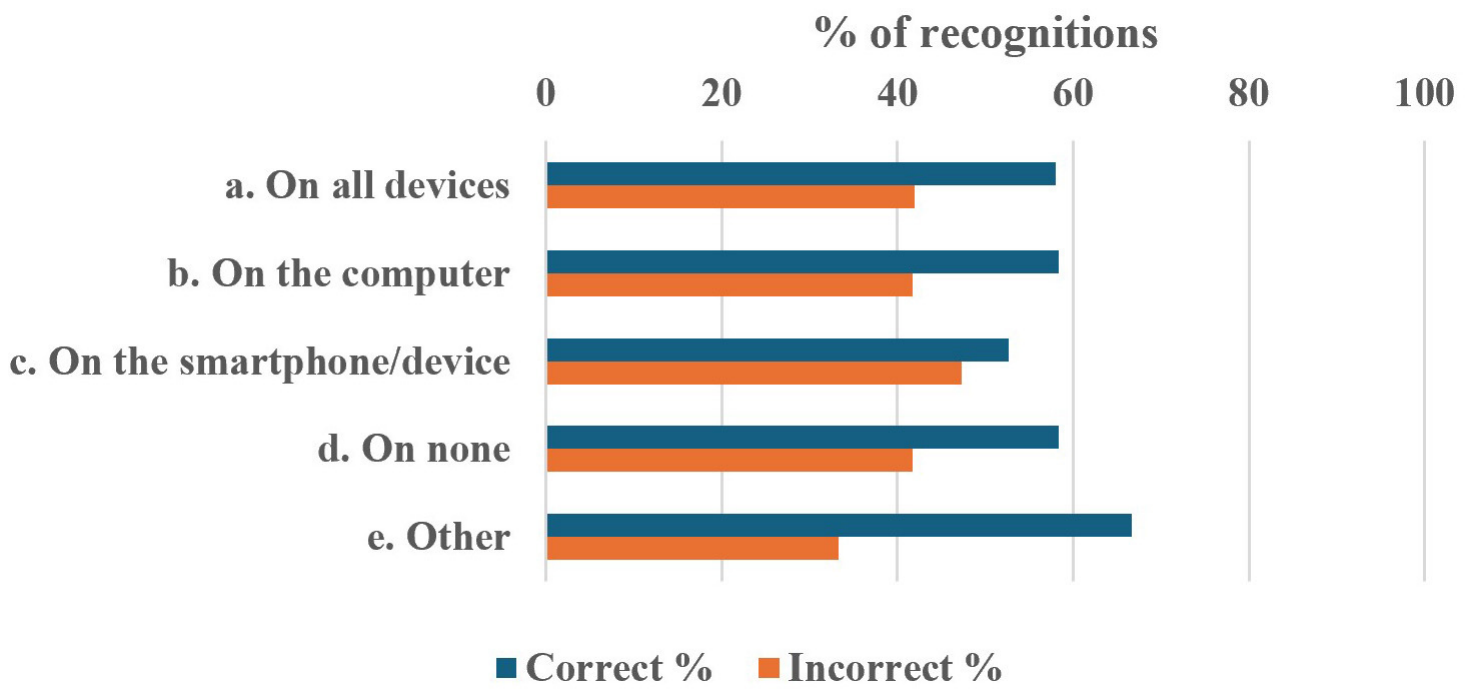
The % of correct recognitions of news veracity (in total) in dependence of cyber hygiene awareness item dealing with enabling security features on different devices.

**Figure 5:**
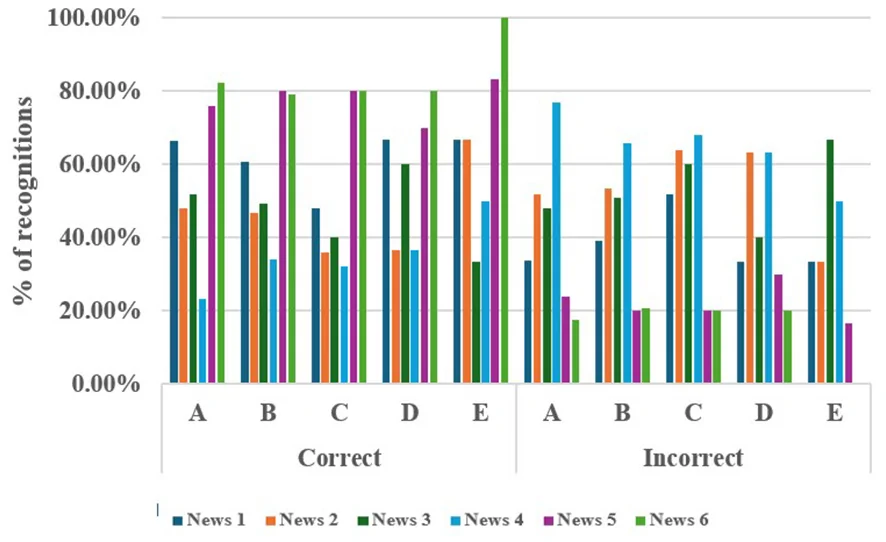
The % of correct recognitions of news veracity (each news) in dependence of cyber hygiene awareness item dealing with enabling security features on different devices. Legend: a. On all devices, b. On the computer, c. On the smartphone/device, d. On none, e. Other.

The same situation is with cyber hygiene awareness item dealing with security checking the files and devices before downloading or using them in terms of news true or fake detecting ability. Regardless of their awareness level, they equally detected news as fake or true correctly, either in general (Figure 6), or for each (Figure 7) of offered fake news.

**Figure 6:**
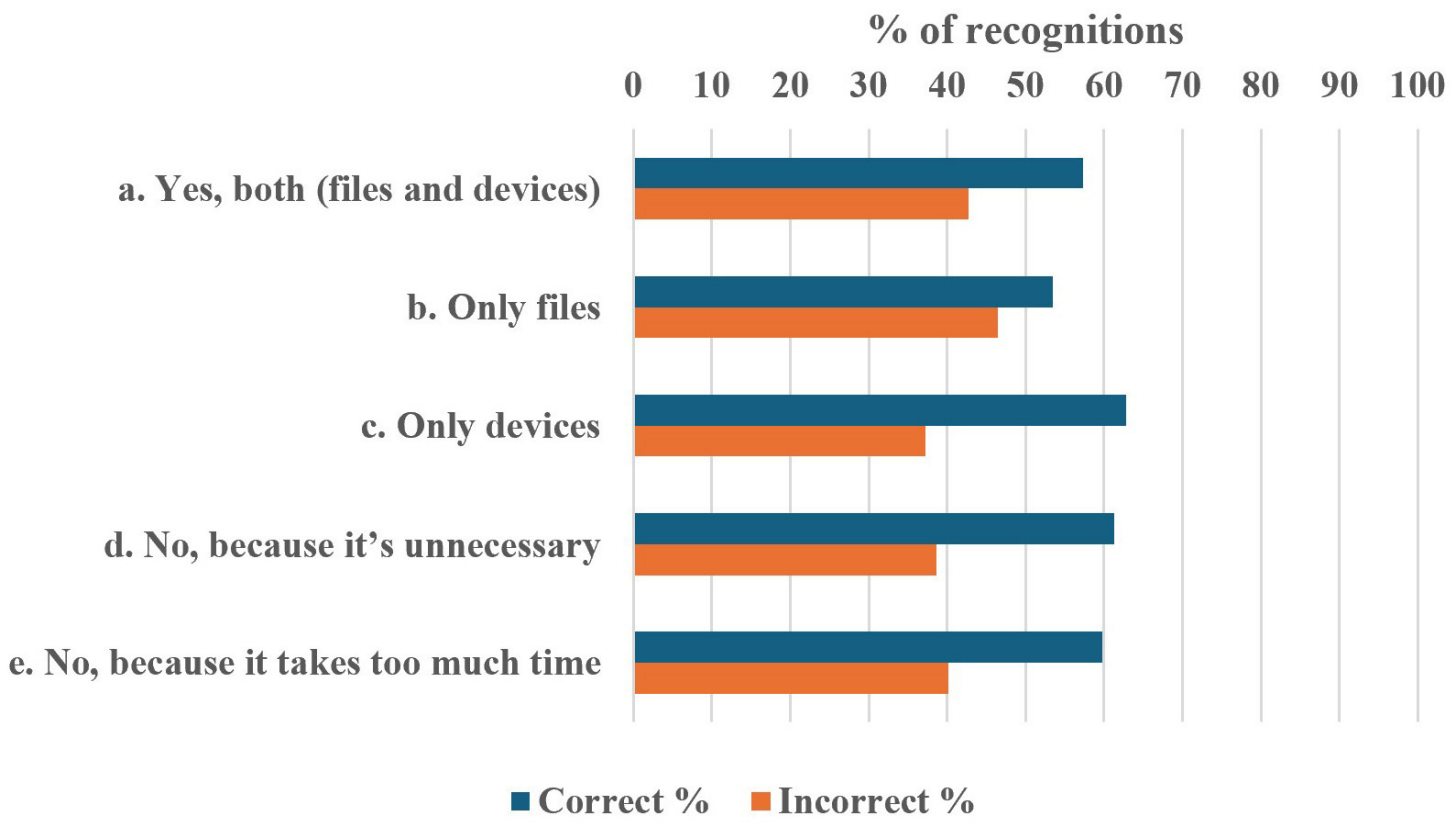
The % of correct recognitions of news veracity (in total) in dependence of cyber hygiene awareness item dealing with security checking the files and devices before downloading or using them.

**Figure 7:**
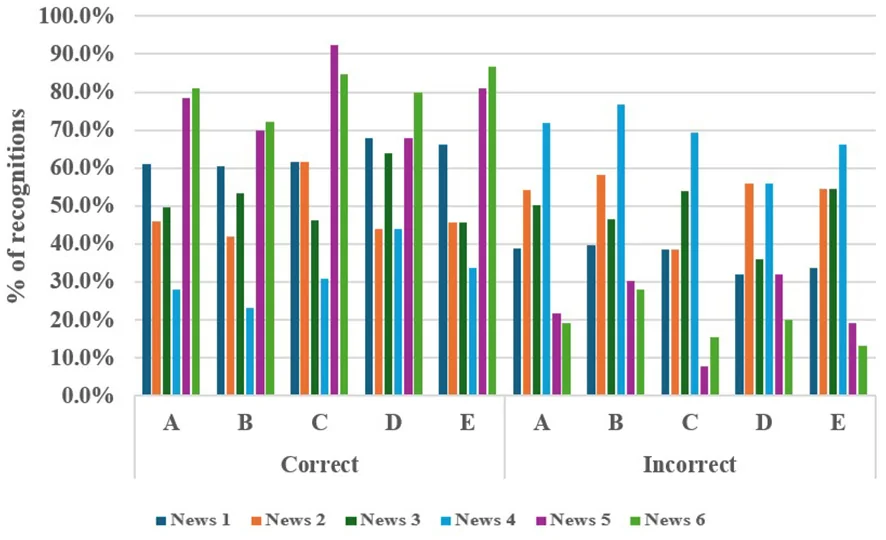
The % of correct recognitions of news veracity (each news) in dependence of cyber hygiene awareness item dealing with security checking the files and devices before downloading or using them. Legend: a. Yes, on both (files and devices), b. Only files, c. Only devices, d. No, because it's unnecessary, e. No, because it takes too much time.

It can be concluded based on the obtained results, how often participants make backup of their data is not related to their news veracity judgement. Graphs given in Figures 8, 9, show that there is almost equal percentage of correct recognitions in general and for each individual news regardless of backup habits.

**Figure 8:**
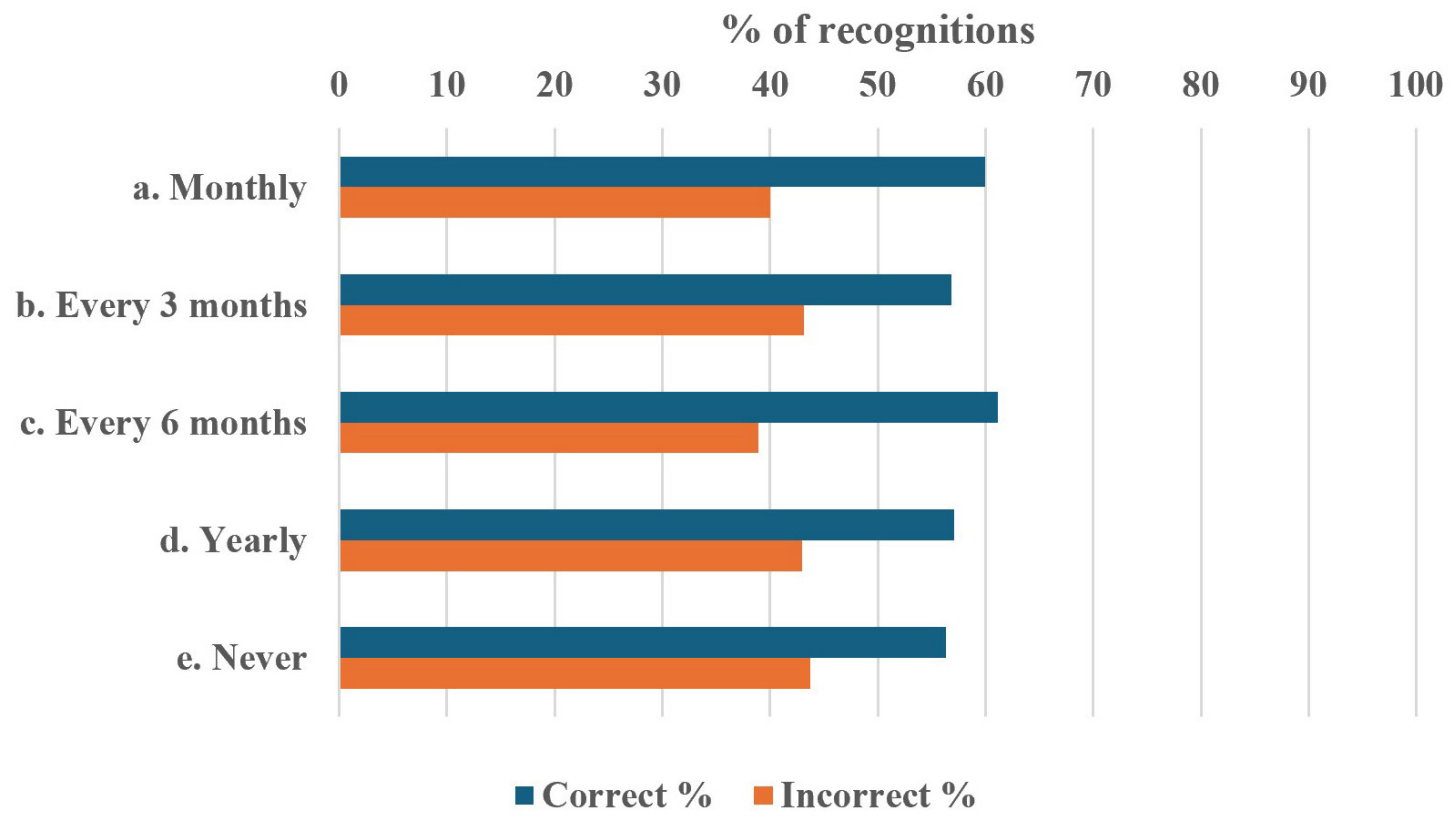
The % of correct recognitions of news veracity (in total) in dependence of cyber hygiene awareness item dealing with backup strategy.

**Figure 9:**
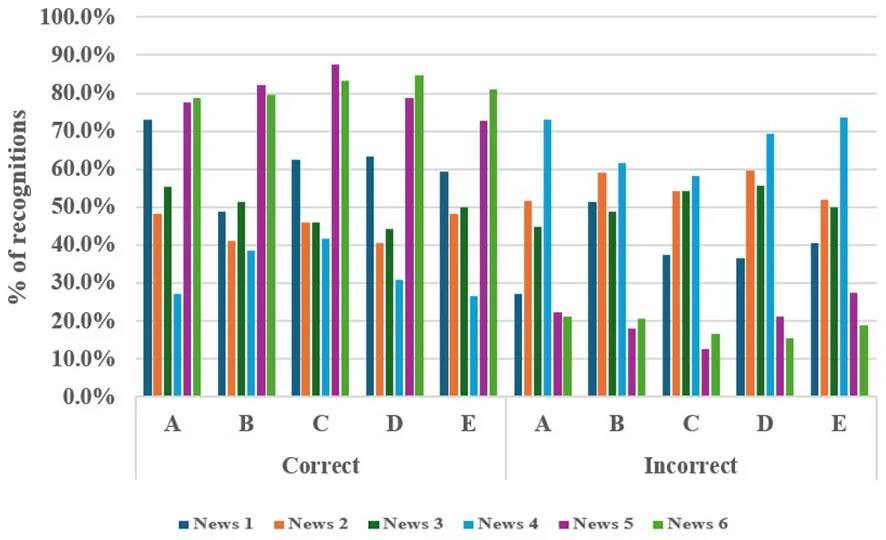
The % of correct recognitions of news veracity (each news) in dependence of cyber hygiene awareness item dealing with backup strategy. Legend: a. Monthly, b. Every 3 months, c. Every 6 months, d. Yearly, e. Never.

The same situation is with participants' cyber hygiene awareness regarding the smart device software updates and its relation to detection of fake or true news. Graphs in Figures 10, 11, show no clear difference in general and for each news in particular in percentage of correct recognitions for all offered four options of software update.

**Figure 10:**
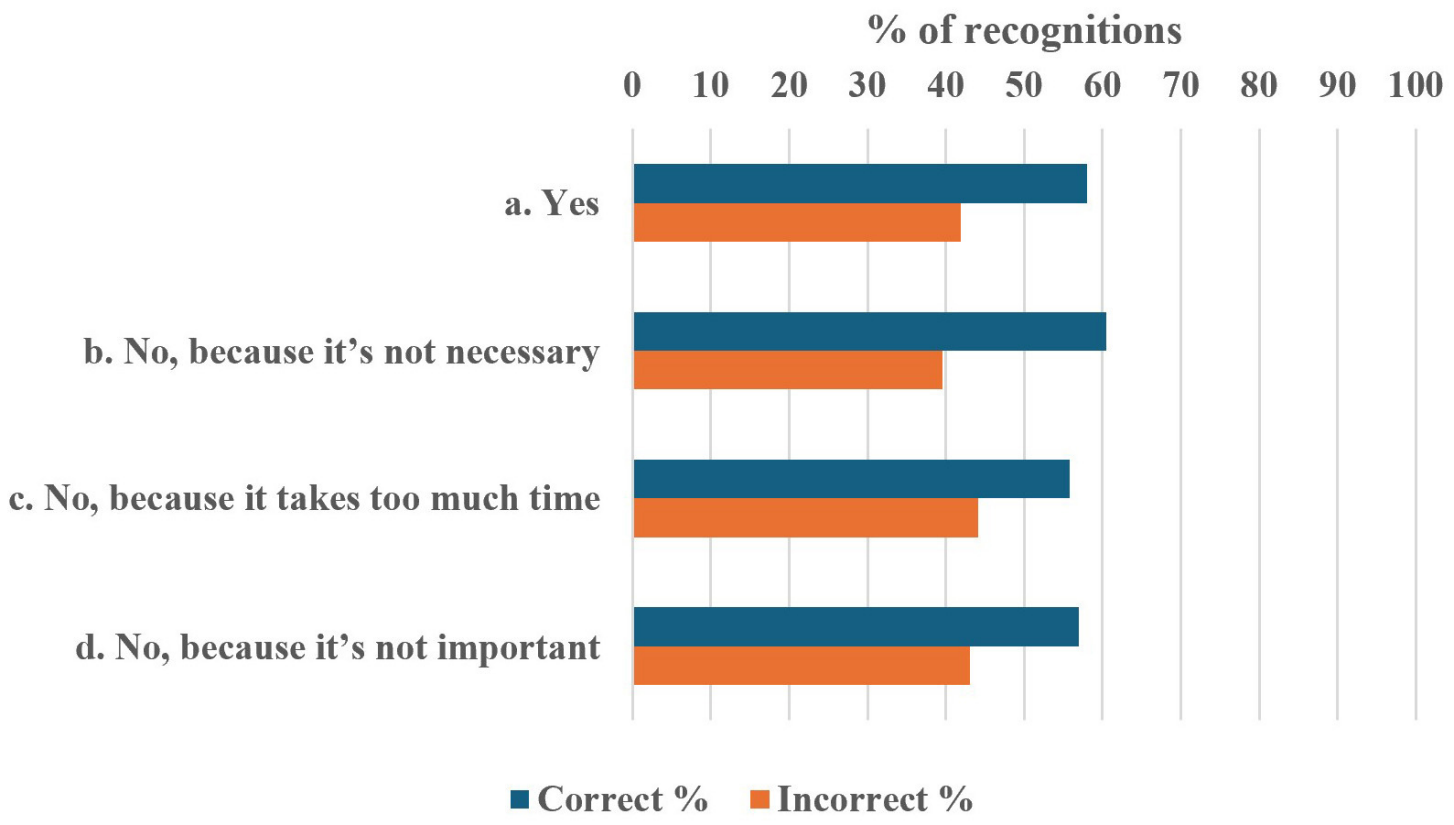
The % of correct recognitions of news veracity (in total) in dependence of cyber hygiene awareness item dealing with smart device software updates.

**Figure 11:**
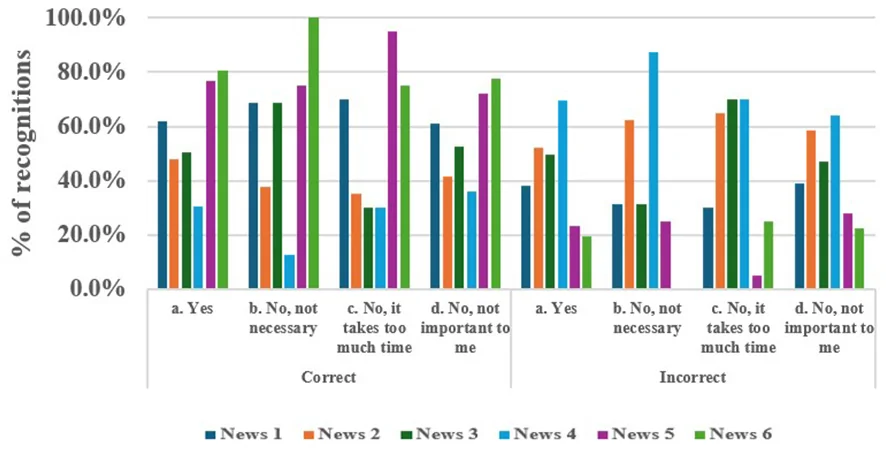
The % of correct recognitions of news veracity (each news) in dependence of cyber hygiene awareness item dealing with smart device software updates.

Similar results are obtained with participants' cyber hygiene awareness regarding the safety of links for software download on computer device and its relation to detection of fake or true news. Graphs (Figures 12, 13) show no clear difference in general and for each news in particular in percentage of correct recognitions for usage of safe links. The obtained results remain the same for participants' cyber hygiene awareness regarding the safety of links for software download on smartphone device and its impact on detection of fake or true news (Figures 14, 15). All these results have been supported by the Chi-square test numbers in Table 3 which show that there is no statistically significant relation between independent and dependent variables. Furthermore, all observed effect sizes were small (Cramér's *V* < 0.20), indicating that any associations between cyber hygiene awareness variables and news veracity judgments were negligible in practical terms, even when statistical significance was approached. Because none of the awareness-related comparisons achieved nominal statistical significance, false-discovery-rate adjustment did not alter the findings. The conclusion of no detectable association remained unchanged.

**Figure 12:**
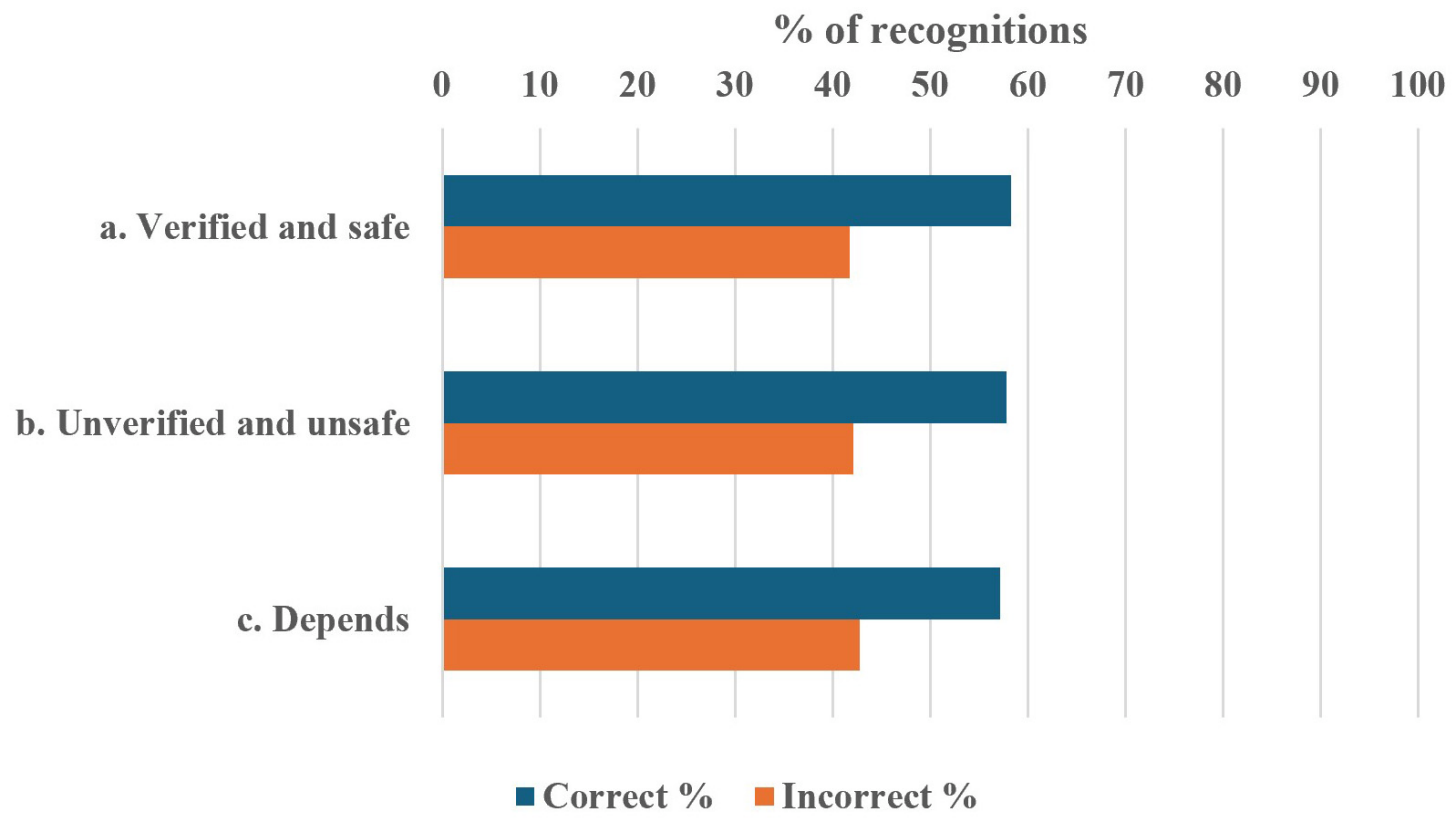
The % of correct recognitions of news veracity (in total) in dependence of cyber hygiene awareness item dealing with the safety of links for software download on computer device.

**Figure 13:**
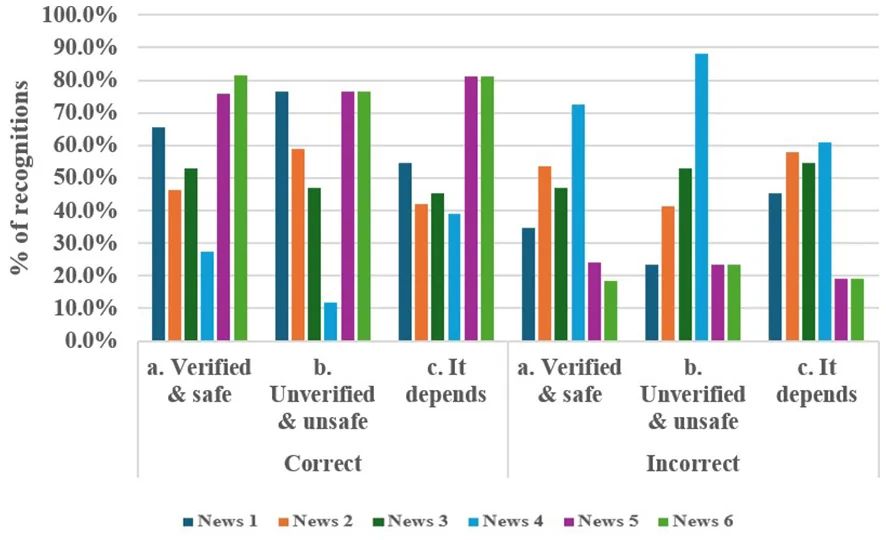
The % of correct recognitions of news veracity (each news) in dependence of cyber hygiene awareness item dealing with the safety of links for software download on computer device.

**Figure 14:**
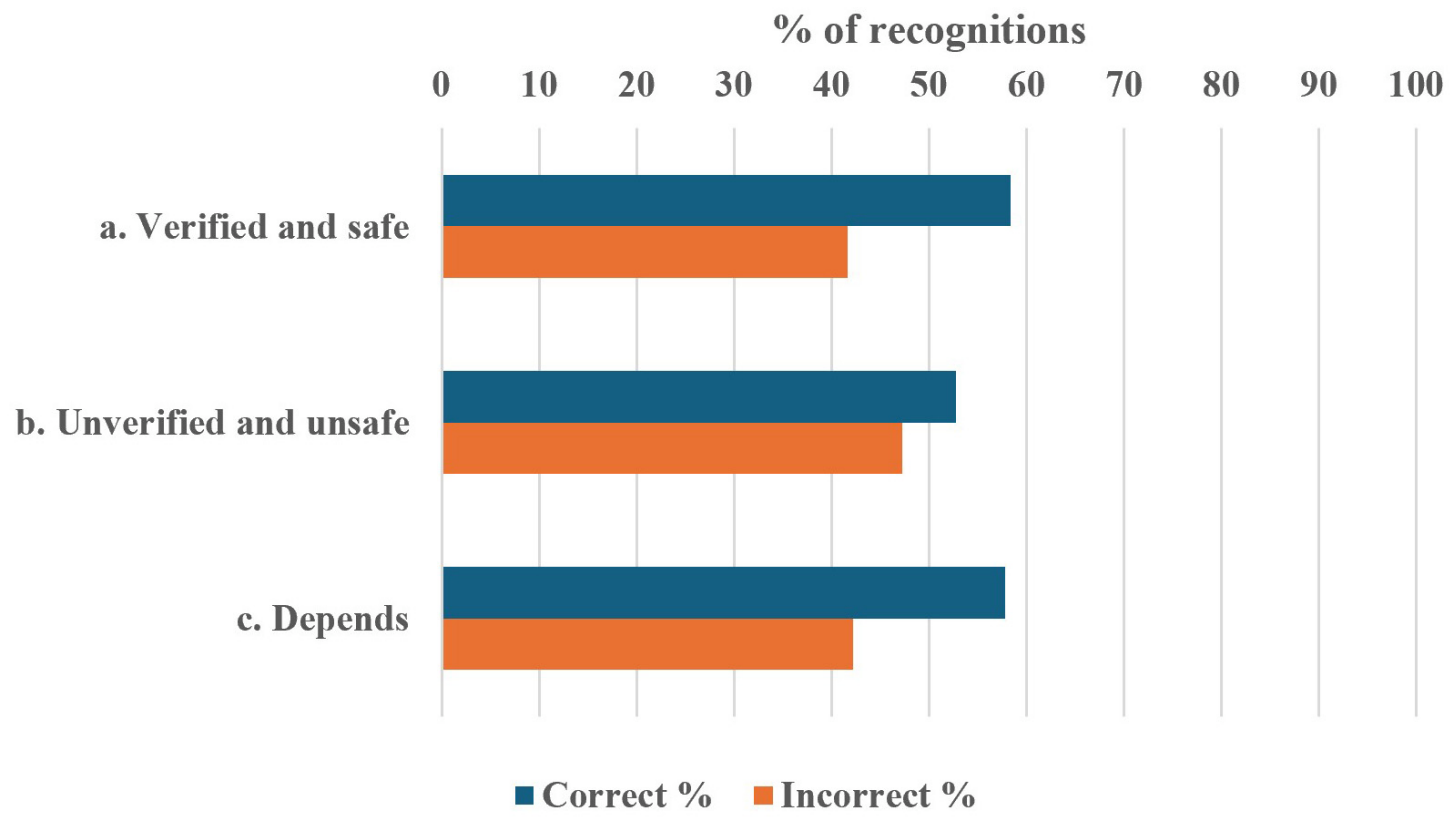
The % of correct recognitions of news veracity (in total) in dependence of cyber hygiene awareness item dealing with the safety of links for software download on smartphone.

**Figure 15:**
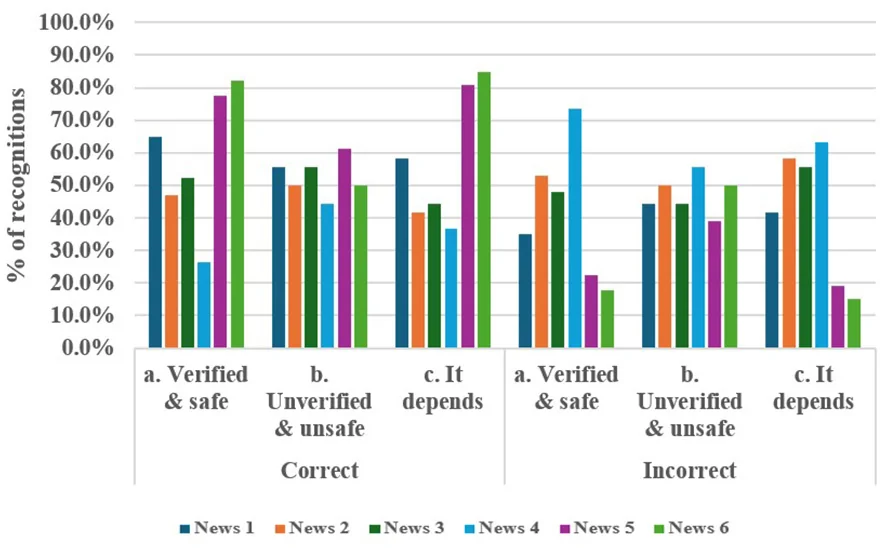
The % of correct recognitions of news veracity (each news) in dependence of cyber hygiene awareness item dealing with the safety of links for software download on smartphone.

**Table 3:** Chi-square test results for cyber hygiene awareness questions and news veracity questions (*N* = 306).

Survey question	News 1		News 2		News 3		News 4		News 5		News 6	
	*χ^2^*-value	*p*-value	*χ^2^*-value	*p*-value	*χ^2^*-value	*p*-value	*χ^2^*-value	*p*-value	*χ^2^*-value	*p*-value	*χ^2^*-value	*p*-value
Q7 (df = 4)	3.46	0.483	3.31	0.508	3.09	0.543	5.56	0.234	1.76	0.779	1.87	0.760
Q20 (df = 4)	0.91	0.923	1.60	0.809	2.77	0.597	4.03	0.402	4.91	0.297	3.82	0.431
Q27 (df = 4)	7.57	0.109	1.40	0.844	1.82	0.768	3.89	0.421	3.32	0.505	0.85	0.932
Q28 (df = 3)	0.80	0.850	2.03	0.566	5.57	0.135	2.98	0.394	4.18	0.242	4.48	0.214
Q29 (df = 2)	4.95	0.101	1.71	0.425	1.64	0.440	6.97	0.201	1.03	0.598	0.25	0.882
Q30 (df = 2)	1.57	0.456	0.74	0.689	1.62	0.444	4.83	0.089	3.33	0.190	12.24	0.200
	**Cramer's** ***V***	**Effect size**	**Cramer's** ***V***	**Effect size**	**Cramer's** ***V***	**Effect size**	**Cramer's** ***V***	**Effect size**	**Cramer's** ***V***	**Effect size**	**Cramer's** ***V***	**Effect size**
Q7	0.11	Small	0.10	Small	0.10	Small	0.13	Small	0.08	Small	0.08	Small
Q20	0.05	Small	0.07	Small	0.10	Small	0.11	Small	0.13	Small	0.11	Small
Q27	0.16	Small	0.07	Small	0.08	Small	0.11	Small	0.10	Small	0.05	Small
Q28	0.05	Small	0.08	Small	0.13	Small	0.10	Small	0.12	Small	0.12	Small
Q29	0.12	Small	0.07	Small	0.07	Small	0.15	Small	0.06	Small	0.03	Small
Q30	0.07	Small	0.05	Small	0.07	Small	0.13	Small	0.10	Small	0.20	Small

To sum up and to give an answer to research question RQ3 dealing with the relation between cyber hygiene awareness and the news veracity judgement, one can conclude that no statistically significant association was observed in the present sample.

### 4.3 The relation between cyber hygiene behavior and the news veracity judgement

The obtained results given in Table 4 (and Supplementary Tables A–I containing percentages) show that for each cyber hygiene behavior question (Q3, Q4, Q5, Q6, Q9, Q10, Q12, Q13, Q14) there is no statistically significant association to the news veracity judgement. Although several comparisons produced non-zero effect sizes, all observed effects remained within the small range. Therefore, the practical significance of the observed relationships between cyber hygiene behavior and news veracity judgments was limited. It can be concluded that regardless of the devices used for Internet usage, who has access to them, and what applications are used via those devices (Q3–Q6) there is no evident difference in the percentage of correct identification of news. The same results are obtained for the habits when creating passwords are in question (Q9–Q10), as well as for data shared through the social networks (Q12), and Wi-Fi usage habits (Q12–Q14). Similarly, none of the behavior-related comparisons achieved nominal statistical significance. Consequently, false-discovery-rate adjustment did not change the interpretation of the results. In order to summarize and to answer the research question RQ4, there is no statistically significant association between cyber hygiene behavior and the news veracity judgement.

**Table 4:** Chi-square test results for cyber hygiene behavior questions and news veracity questions (*N* = 306).

Survey question	News 1		News 2		News 3		News 4		News 5		News 6	
	*χ^2^*-value	*p*-value	*χ^2^*-value	*p*-value	*χ^2^*-value	*p*-value	*χ^2^*-value	*p*-value	*χ^2^*-value	*p*-value	*χ^2^*-value	*p*-value
Q3 (df = 3)	3.70	0.296	1.09	0.779	2.08	0.555	5.39	0.145	1.07	0.784	3.04	0.386
Q4 (df = 4)	5.76	0.218	7.11	0.130	10.09	0.309	11.72	0.200	4.40	0.354	5.14	0.273
Q5 (df = 4)	2.56	0.634	6.23	0.183	6.69	0.153	7.77	0.100	10.31	0.306	18.83	0.100
Q6 (df = 3)	0.88	0.831	2.92	0.405	2.92	0.405	7.00	0.072	7.00	0.072	3.94	0.268
Q9 (df = 4)	3.04	0.552	3.39	0.494	4.20	0.380	3.22	0.522	4.26	0.372	2.99	0.560
Q10 (df = 4)	1.33	0.856	5.42	0.247	3.75	0.441	3.40	0.493	0.32	0.988	7.07	0.132
Q12 (df = 5)	8.33	0.139	4.27	0.511	5.51	0.357	5.39	0.370	4.58	0.469	8.31	0.140
Q13 (df = 3)	3.47	0.325	2.60	0.458	2.08	0.556	0.91	0.823	2.49	0.477	4.67	0.198
Q14 (df = 3)	0.32	0.957	0.32	0.956	2.78	0.427	0.69	0.875	0.85	0.838	1.66	0.647
	**Cramer's** ***V***	**Effect size**	**Cramer's** ***V***	**Effect size**	**Cramer's** ***V***	**Effect size**	**Cramer's** ***V***	**Effect size**	**Cramer's** ***V***	**Effect size**	**Cramer's** ***V***	**Effect size**
Q3	0.11	Small	0.06	Small	0.08	Small	0.13	Small	0.06	Small	0.10	Small
Q4	0.15	Small	0.17	Small	0.20	Small	0.20	Small	0.13	Small	0.14	Small
Q5	0.09	Small	0.15	Small	0.15	Small	0.16	Small	0.19	Small	0.25	Medium
Q6	0.05	Small	0.10	Small	0.10	Small	0.15	Small	0.15	Small	0.11	Small
Q9	0.10	Small	0.10	Small	0.12	Small	0.10	Small	0.12	Small	0.09	Small
Q10	0.07	Small	0.13	Small	0.11	Small	0.11	Small	0.03	Small	0.15	Small
Q12	0.16	Small	0.12	Small	0.13	Small	0.13	Small	0.12	Small	0.16	Small
Q13	0.12	Small	0.11	Small	0.09	Small	0.06	Small	0.10	Small	0.14	Small
Q14	0.03	Small	0.03	Small	0.10	Small	0.05	Small	0.05	Small	0.07	Small

### 4.4 The relation between cyber hygiene knowledge and the news veracity judgement

In order to analyze the relation between cyber hygiene knowledge and the news veracity judgement, we performed Chi-squared test and reported the results in Table 5. The results show that statistically significant associations were observed for some specific cyber hygiene knowledge–news pairs. However, given the large number of item-level comparisons and the absence of a consistent pattern across the six news stimuli, these individual associations should be interpreted as exploratory and hypothesis-generating rather than as evidence of a systematic relationship. For example, knowledge of HTTPS (Q17), private search (Q18), phishing (Q21), two-factor authentication (Q22), botnets (Q23), and ransomware (Q24) showed statistically significant associations with the evaluation of news 2 and/or news 3. However, these associations were not consistently observed across the remaining stimuli. The findings therefore do not provide evidence of a stable relationship between cyber hygiene knowledge and general news-veracity judgment performance. Also, effect sizes were uniformly small (Cramér's *V* ≤ 0.21), suggesting limited practical importance of these associations.

**Table 5:** Chi-square test results for cyber hygiene knowledge questions and news veracity questions (*N* = 306).

Survey question	News 1		News 2		News 3		News 4		News 5		News 6	
	*χ^2^*-value	*p*-value	*χ^2^*-value	*p*-value	*χ^2^*-value	*p*-value	*χ^2^*-value	*p*-value	*χ^2^*-value	*p*-value	*χ^2^*-value	*p*-value
Q15 (df = 1)	1.69	0.192	0.03	0.849	0.84	0.359	**3.78**	**0.050**	0.86	0.352	1.53	0.216
Q16 (df = 1)	0.01	0.969	3.63	0.057	3.37	0.066	0.69	0.405	0.04	0.947	0.70	0.400
Q17 (df = 1)	2.87	0.090	**7.43**	**0.006**	**10.16**	**0.001**	0.08	0.772	0.04	0.832	0.01	0.985
Q18 (df = 1)	0.10	0.742	**4.08**	**0.043**	**4.70**	**0.030**	0.60	0.438	0.08	0.768	0.94	0.331
Q19 (df = 1)	0.01	0.976	0.26	0.610	1.17	0.279	1.14	0.286	2.06	0.151	0.19	0.656
Q21 (df = 1)	**5.65**	**0.017**	**3.81**	**0.050**	**3.85**	**0.050**	2.63	0.104	0.03	0.853	**5.61**	**0.018**
Q22 (df = 1)	2.19	0.139	**3.75**	**0.050**	**7.67**	**0.006**	2.53	0.112	0.21	0.641	0.25	0.611
Q23 (df = 1)	1.71	0.191	**5.42**	**0.020**	**9.89**	**0.002**	0.07	0.789	3.73	0.053	0.17	0.677
Q24 (df = 1)	3.16	0.075	**7.39**	**0.007**	**14.27**	**0.000**	**3.95**	**0.047**	**4.04**	**0.044**	0.58	0.455
Q25 (df = 1)	1.07	0.301	0.45	0.500	0.01	0.984	0.30	0.581	1.46	0.226	0.07	0.784
Q26 (df = 1)	1.45	0.227	0.40	0.524	0.81	0.369	0.76	0.381	3.32	0.068	2.66	0.102
	**Cramer's** ***V***	**Effect size**	**Cramer's** ***V***	**Effect size**	**Cramer's** ***V***	**Effect size**	**Cramer's** ***V***	**Effect size**	**Cramer's** ***V***	**Effect size**	**Cramer's** ***V***	**Effect size**
Q15	0.07	Small	0.01	Small	0.05	Small	0.11	Small	0.05	Small	0.07	Small
Q16	0.02	Small	0.10	Small	0.10	Small	0.04	Small	0.00	Small	0.04	Small
Q17	0.09	Small	0.16	Small	0.18	Small	0.01	Small	0.01	Small	0.00	Small
Q18	0.01	Small	0.11	Small	0.12	Small	0.04	Small	0.01	Small	0.05	Small
Q19	0.01	Small	0.02	Small	0.06	Small	0.06	Small	0.08	Small	0.02	Small
Q21	0.13	Small	0.11	Small	0.11	Small	0.09	Small	0.01	Small	0.13	Small
Q22	0.08	Small	0.11	Small	0.15	Small	0.09	Small	0.02	Small	0.03	Small
Q23	0.07	Small	0.13	Small	0.18	Small	0.01	Small	0.11	Small	0.02	Small
Q24	0.10	Small	0.15	Small	0.21	Medium	0.11	Small	0.11	Small	0.04	Small
Q25	0.06	Small	0.04	Small	0.01	Small	0.03	Small	0.07	Small	0.01	Small
Q26	0.07	Small	0.03	Small	0.05	Small	0.05	Small	0.10	Small	0.09	Small

To evaluate the robustness of significant findings, the Benjamini-Hochberg false-discovery-rate procedure was applied to the 60 cyber-hygiene knowledge comparisons. Following adjustment, only three associations remained statistically significant: Q17-news 3, Q23-news 3, and Q24-news 3. All remaining nominally significant findings became non-significant after adjustment. Because the surviving effects were confined to a single news stimulus and were not replicated across the remaining items, the overall interpretation remained unchanged, namely that no consistent relationship between cyber hygiene knowledge and news-veracity judgment was observed.

Although statistically significant associations between certain cyber hygiene variables and news veracity judgement were observed for a subset of individual news items, these effects were not consistent across all items and did not exhibit a clear pattern. Given that veracity judgement was assessed across multiple stimuli, a stable relationship would be expected to produce consistent effects. The observed sporadic significance therefore suggests that these associations may be driven by item-specific characteristics rather than reflecting a general relation of cyber hygiene and news veracity judgement. Consequently, no definitive conclusion regarding a systematic relationship between these variables can be drawn.

To avoid the misleading conclusions, the authors believe that additional tests need to be conducted before concluding if the relation between cyber hygiene knowledge and the news veracity judgement exists. Therefore, the answer to the research question RQ5 dealing with the relation between cyber hygiene knowledge and the news veracity judgement is that such connection does not show consistent pattern and needs to be further analyzed.

### 4.5 Summary of results

In total, 162 item-level Chi-square tests were conducted (6 age-related, 6 gender-related, 36 awareness-related, 54 behavior-related, and 60 knowledge-related comparisons). Significant associations generally exhibited small effect sizes (*V* = 0.12–0.25), suggesting that, although statistically detectable, the practical importance of these relationships is limited. For gender, the association with news 2 remained statistically significant after false-discovery-rate adjustment ]χ^2^(1, *N* = 306) = 8.12, *p* = 0.004, Cramér's *V* = 0.16, 95% CI (0.05, 0.28)]. For the cyber-hygiene knowledge dimension, three associations remained statistically significant after FDR adjustment: Q17 with News 3 [χ^2^(1, *N* = 306) = 10.16, *p* = 0.001, *V* = 0.18, 95% CI (0.07, 0.29)], Q23 with News 3 [χ^2^(1, *N* = 306) = 9.89, *p* = 0.002, *V* = 0.18, 95% CI (0.07, 0.29)], and Q24 with News 3 [χ^2^(1, *N* = 306) = 14.27, *p* < 0.001, *V* = 0.22, 95% CI (0.10, 0.33)]. These intervals indicate uncertainty around the observed effect-size estimates and reinforce the interpretation that the detected associations were small in magnitude. The summary of results is given in Table 6.

**Table 6:** Research questions and answers.

Research question (RQ)	Answer
Is there a relation between gender and the judgement of news veracity?	Partial association with veracity judgment tendency
Is there a relation between age and the judgement of news veracity?	No consistent association observed
Is there a relation between cyber hygiene awareness and the judgement of news veracity?	No statistically detectable association observed
Is there a relation between cyber hygiene behavior and the judgement of news veracity?	No statistically detectable association observed
Is there a relation between cyber hygiene knowledge and the judgement of news veracity?	Limited and stimulus-specific associations; no consistent pattern observed

## 5 Discussion

The findings presented in this paper suggest that cyber hygiene awareness, behavior, and knowledge levels are not significantly associated with survey participants' news veracity judgement. In addition to the lack of a consistent pattern of statistical significance, the observed effect sizes were generally small. This indicates that even where associations were detected, their practical importance was limited. Taken together, the findings do not provide evidence of a consistent overall relationship between the measured cyber hygiene dimensions and participants' news-veracity judgments.

One plausible interpretation is that cyber hygiene and judgement of news veracity rely on partially different cognitive processes. Cyber hygiene awareness (e.g., backups, link, etc.), behaviors (e.g., updating passwords, enabling two-factor authentication, etc.), or knowledge (e.g., identifying what is virtual private network (VPN), recognizing phishing links, etc.) are procedural and habit-based; they are often learned as concrete rules and reinforced through repetition (2, 60, 63). In contrast, judging the veracity of news requires evaluative reasoning. In other words, it assesses credibility, interpreting context, recognizing rhetorical manipulation, and applying domain knowledge (64, 65). A survey participant may competently follow security routines without engaging in deeper analytical scrutiny of informational content. This helps explain why individuals who are “secure users” are not necessarily “critical readers,” and it suggests that prior assumptions in the literature may have implicitly conflated technical security literacy with epistemic or media literacy (66).

Another explanation is methodological and contextual. Many previous studies infer a relationship indirectly by assuming that digitally literate or experienced users should be better at identifying fake news, yet they often measure digital competence broadly rather than testing fake news detection directly (67). This survey, by empirically testing detection performance, indicates that the relationship may be weaker than theoretically expected or conditional on other variables such as education level, prior beliefs, cognitive reflection, or confirmation bias. In practice, fake news susceptibility is strongly shaped by psychological and social factors, e.g., motivated reasoning, emotional reactions, and trust in sources (68), which cyber hygiene training may not directly address. Therefore, the absence of a significant association does not imply that cyber hygiene is unimportant; rather, it suggests that the present findings do not provide evidence that online safety behaviors are associated with lower vulnerability to fake news. Interventions aimed at fake news resilience may need to focus specifically on critical thinking, source evaluation, and cognitive bias awareness rather than relying on general cyber hygiene awareness, behavior, and knowledge programs (69).

The findings have broader implications for digital wellbeing and quality of life because they are consistent with the view that technical cyber hygiene and epistemic evaluation of information may represent distinct dimensions of digital competence. Individuals may follow recommended cybersecurity practices and successfully protect their devices and online accounts while still experiencing difficulties in evaluating the credibility of information. Consequently, digital safety should not be assumed to imply resilience to fake news. While cyber hygiene is primarily concerned with technical protection, news veracity judgment appears to depend more strongly on evaluative processes such as critical thinking, source assessment, and reflective reasoning (69, 70). The present findings are therefore consistent with the view that improving digital wellbeing requires complementary but distinct educational approaches, including cyber hygiene education for technical protection and media-literacy or critical-thinking interventions for information evaluation (71).

This distinction may be relevant across different stages of life. In the context of aging, older adults may successfully adopt cybersecurity practices while remaining vulnerable to misleading health, financial, or public-safety information, indicating that technical competence does not necessarily translate into interpretive competence (72, 73). Similarly, younger users frequently described as “digital natives” may demonstrate high levels of technological proficiency yet still rely on superficial cues such as popularity indicators, emotional tone, or influencer status when evaluating information credibility (74–76). Consistent with previous research, the current results suggest that vulnerability to fake news may depend more strongly on cognitive, psychological, and social factors than on technical competence alone (77–79). Although no age-related differences in news veracity judgment were observed in the present study, this finding should be interpreted cautiously given the strong overrepresentation of younger participants in the sample.

Similar considerations apply to organizational environments, where employees may comply with cybersecurity practices and security protocols while still encountering difficulties in evaluating information reliability (13, 80). Modern workplaces increasingly depend on accurate interpretation of reports, communications, and online information, suggesting that cybersecurity competence and information-evaluation competence should not be treated as interchangeable (81, 82). Taken together, the findings are consistent with the view that functional independence in the digital environment is multidimensional: being technically secure does not necessarily imply being epistemically secure. Consequently, interventions aimed at strengthening digital resilience may benefit from addressing technical protection skills and information-evaluation skills as related but distinct competencies (83, 84).

The gender difference in news veracity judgment adds an important layer to the interpretation of our results. Women in the study demonstrated greater skepticism, evaluating more news items as false compared with men. This suggests that affective and cognitive dispositions, such as caution, distrust, or risk aversion, may influence news veracity judgments independently of technical skills such as cyber hygiene. However, this pattern should not be interpreted as evidence of superior detection performance. While female participants were more likely to classify news items as false, sensitivity-specificity analysis demonstrated a substantial asymmetry between identification of false items (79.2%) and true items (47.2%). Furthermore, the negative response criterion (*c* = −0.23) indicated a systematic tendency toward judging items as false. Consequently, the observed gender differences appear more consistent with differences in response tendency than with differences in veracity discrimination ability. Increased skepticism may provide some protection against misleading content and support safer decision making (43), but excessive distrust may also be associated with increased cognitive burden and difficulties in information-rich environments (85). Distinguishing between veracity discrimination and response bias is therefore essential. Future research should explicitly account for these dimensions, for example by separately examining sensitivity, specificity, balanced accuracy, and other response-bias indicators, in order to determine whether observed demographic differences reflect genuine differences in performance or differences in evaluative tendencies.

Within the broader framework of cyber hygiene, these findings reinforce the idea that procedural safety behaviors do not capture the full spectrum of online resilience. While both men and women may follow security protocols equally, women's higher evaluative caution reflects a psychological dimension of digital competence that is not addressed by standard cyber hygiene practices. Interventions aiming to improve overall digital wellbeing should therefore consider not only technical and epistemic skills but also the cognitive and emotional factors, including gender differences in trust and vigilance, which shape online behavior, information judgment, and ultimately life satisfaction.

Because the study employed a cross-sectional observational design, the findings should be interpreted as associations rather than causal relationships. The study is also exploratory and based on a convenience sample; therefore, the findings should not be interpreted as population-representative estimates. Future research using larger, more diverse samples and longitudinal or experimental designs could examine whether the observed relationships are robust and clarify their underlying mechanisms.

## 6 Conclusion

### 6.1 Concluding remarks

The most existing literature is non-empirical and assumes the positive relationship between cyber hygiene and the accurately judge news veracity, i.e., one detects fake news with greater accuracy if they have better cyber hygiene level. In order to test this assumption and achieve the primary goal of the paper, i.e., to explore the relationship between cyber hygiene and news veracity judgement, we have conducted a survey study containing 30 questions related to estimation of cyber hygiene awareness, behavior, and knowledge level and 10 questions related to fake news with 6 news being offered for veracity determination.

The results indicate no consistent association between cyber hygiene dimensions and participants' veracity judgments of the selected news items. Because the fake-news module contained more true than false items and was not designed to separate discrimination ability from response tendency, the findings should be interpreted as reflecting patterns of news veracity judgment rather than a definitive measure of balanced fake-news detection accuracy. Our study also showed the existence of gender relation to the news veracity judgement, in terms of higher skepticism at women in comparison to men, and no associations or clear patterns when it comes to age groups. It is important to stress that the results of the study should be considered indicative rather than representative since the study is exploratory in nature. However, the absence of statistical significance should not automatically be interpreted as evidence that no relationship exists. Limited power, measurement limitations, restricted variability, sampling bias, and stimulus characteristics may all contribute to null findings. The conclusions should therefore be framed as evidence that no consistent association was detected under the present sampling, measurement, and analytical conditions.

The contributions of this study are two-fold: firstly, this is the first empirical and comprehensive study to test the relationship between cyber hygiene awareness, behavior, and knowledge and the news veracity judgement. Secondly, rather than demonstrating the expected positive relationship, the study found no consistent association between cyber hygiene dimensions and news veracity judgments.

The interpretation of findings, limitations, and future work are given in the following subsections.

### 6.2 Interpretation of findings

The obtained results have several interpretations, particularly in relation to how cyber hygiene and news veracity judgement are conceptualized. The findings do not demonstrate a consistent overall relationship between the measured cyber hygiene dimensions and news-veracity judgments in the present sample. However, the observed pattern raises the possibility that these constructs may not be as straightforward as often assumed in the literature.

#### 6.2.1 Theoretical Interpretation

The results are consistent with the possibility that cyber hygiene behaviors and the news veracity judgement may represent distinct dimensions of digital competence. While previous research has sometimes considered cybersecurity practices, digital literacy, and fake news resilience as part of a unified skill set, the lack of a consistent relationship observed in this study suggests that these domains should not automatically be treated as interchangeable. In particular, cyber hygiene appears to technical and procedural practices, whereas news veracity judgement may rely more on evaluative and cognitive processes. These findings point to the need for more precise conceptualization and operationalization of digital competence in future research. This interpretation remains tentative and should be examined using validated measures specifically designed to assess these potentially distinct competencies.

The findings also indicate that cyber hygiene-related knowledge and behaviors should not be assumed, on the basis of the present data, to provide a direct indicator of individuals' ability to evaluate the veracity of online information. Because the study did not assess educational interventions, policy measures, or organizational outcomes, no conclusions regarding the effectiveness of particular educational, policy, or organizational approaches can be drawn.

#### 6.2.2 Research

The study highlights the importance of directly measuring news veracity judgement rather than inferring it from general digital competence or cybersecurity behaviors. The results also suggest that future research should investigate additional factors that may influence news veracity judgement, such as cognitive and psychological variables, and should employ more robust measurement instruments and analytical approaches. Future studies should also use balanced and psychometrically validated news-veracity stimuli, larger and more diverse samples, and analytical approaches capable of examining multiple predictors simultaneously.

Overall, the present findings do not support treating cybersecurity behaviors and news-veracity judgment as interchangeable constructs. Rather, the absence of a consistent overall relationship in the present sample provides a basis for further investigation of whether technical cyber hygiene and epistemic evaluation represent related but distinct dimensions of digital competence. This interpretation should be regarded as hypothesis-generating rather than as an established causal or theoretical conclusion.

### 6.3 Limitations

As any other, this study has certain limitations which need to be tackled in future research activities. The limitation is that the results may be affected by the participant's demographics and sample size. In other words, the absence of relation between cyber hygiene and the news veracity judgement accurately may be affected by the demographics, and the findings might be different if the study is conducted with different participants (age, culture, gender, etc.). This leads to an open research item for future research, i.e., conducting this survey among participants with different contexts such as gender, age, education, affiliation, culture, etc. to gain clearer picture of situation regarding the connection of cyber hygiene and news veracity judgement.

The study was conducted in Bosnia and Herzegovina, and we acknowledge that cultural, social, and political contexts can shape how individuals respond to survey questions. In particular, factors such as levels of media trust, exposure to fake news, and prevailing digital literacy practices may influence participants' perceptions and veracity judgement. While our survey was designed to be anonymous and non-sensitive, the local context remains an important consideration when interpreting the findings.

We also acknowledge that the precision and stability of subgroup estimates may be affected by the limited number of participants in the older age categories. A limitation of the study concerns the age distribution of the sample. While five age categories were examined, the oldest age group (50+ years) included only 10 participants, resulting in several expected frequencies below recommended thresholds for chi-square analyses. Although supplementary analyses using collapsed age categories produced substantively similar conclusions, age-related findings should be interpreted cautiously and verified in future studies with more balanced age-group representation.

Also, we point out that the result could have been different in dependence of the sample size, which needs to be addressed by the following studies. The study was designed as an exploratory analysis of associations within the obtained sample rather than as a population-representative survey. The convenience sampling approach and the resulting demographic composition, particularly the strong overrepresentation of younger participants, limit external validity and population-level generalization. Future research should employ larger and stratified probability-based samples to enhance external validity and enable more detailed subgroup analyses across demographic categories such as age, gender, and education level.

The use of a convenience sampling strategy introduces potential sampling bias, as participants were recruited via online platforms rather than through probability-based sampling. This, together with the strong overrepresentation of younger adults, limits the generalizability of the findings to the wider population of Bosnia and Herzegovina and constrains the interpretation of age-related effects.

Also, limitation is that news veracity was operationalized as a binary true/false judgment. Although this simplified classification facilitated exploratory analysis, real-world information frequently exists on a continuum of credibility and may include biased, misleading, or selectively framed content that is not strictly false. Furthermore, the binary format does not capture the distinction between factual accuracy and other dimensions of information quality, such as source credibility, framing, or contextual completeness. Future research should therefore investigate more nuanced measures of information evaluation, including source credibility, framing effects, informational bias, and critical assessment of partially accurate content.

Data were collected through online distribution via social media platforms in the Bosnian language, resulting in a convenience sample. Due to the voluntary and anonymous nature of participation, a formal response rate could not be calculated, and non-response bias cannot be statistically assessed. Although the questionnaire was designed to be concise and accessible and participation was available during the data-collection period, these measures cannot rule out self-selection or non-response bias. To reduce potential bias, the questionnaire was kept concise and accessible, and data collection was open for a sufficient period to encourage participation from a broad range of respondents. Nevertheless, the possibility of self-selection bias should be considered when interpreting the findings, and future research should employ probability-based sampling strategies to improve representativeness and allow for systematic assessment of non-response effects.

In addition, the collected data was based on self-reported statistics (i.e., participants were asked to complete the survey). Although a direct validation of the collected responses was avoided, the discussion with the experts and participants provided the understanding of the questions and the responses' validity. Participants were not informed that the final purpose of the survey was to test the relation between cyber hygiene outcomes (i.e., behavior, awareness, knowledge) and news veracity judgement, since it would pump-up their answers. Thus, in the following period, there should be more conducted studies that will deal with the testing of the relation between the cyber hygiene knowledge, awareness, and behavior and news veracity judgement.

Several additional limitations of this study should be acknowledged. The results might be affected by the selection of fake news to be tested. In other words, if different news were selected to be tested in terms of being true or fake, there is a comprehensible possibility that it would affect the final findings. Therefore, the results of the study are affected by fake news items selection. Also, the results might have been affected by the news stimuli order, i.e., the fact that the news were presented in a fixed order to all participants. Consequently, potential order effects, such as fatigue, learning, priming, or response-set formation, cannot be excluded. Future studies should randomize stimulus presentation to reduce the possibility that responses are influenced by item position rather than stimulus content. When it comes to cyber hygiene questionnaire part, the survey has been used for several experimental studies since 2022, but it may be also a cause of such obtained results.

The fake-news module contained four true and two false items, creating an imbalance that limits interpretation of the measure as a pure indicator of fake-news detection accuracy. Sensitivity-specificity analysis showed higher sensitivity (79.2%) than specificity (47.2%), indicating a tendency among participants to classify items as fake. However, a further limitation is that the imbalance between true (*n* = 4) and false (*n* = 2) news items prevents clear separation of detection ability from response bias. Participants with a general tendency to classify news as false may appear more successful on false items while simultaneously misclassifying true items. Consequently, the instrument should be interpreted as measuring veracity judgment behavior rather than balanced detection accuracy. Future studies should employ balanced item sets containing equal numbers of true and false stimuli and should report participant-level sensitivity, specificity, balanced accuracy, and signal-detection metrics. Because familiarity, plausibility, emotional salience, source credibility, and prior knowledge were not formally measured, some observed differences may reflect stimulus-specific characteristics rather than veracity alone.

An additional limitation concerns the large number of item-level statistical comparisons performed in the study. Because multiple Chi-square tests were conducted across cyber hygiene items and individual news stimuli, some statistically significant results may occur by chance when using a nominal significance threshold of *p* ≤ 0.05. Therefore, the statistically significant associations identified at the individual-item level should be regarded as exploratory and hypothesis-generating rather than as confirmatory evidence of systematic relationships. This consideration is particularly important for the cyber hygiene knowledge results, where statistically significant associations were observed for some specific knowledge–news pairs but no consistent pattern was identified across all six news stimuli. The interpretation therefore places greater emphasis on the overall pattern and magnitude of associations than on isolated *p*-values. Also, because each participant evaluated all six stimuli, observations were not statistically independent. The present exploratory analyses did not explicitly model within-participant dependence. Future studies should employ mixed-effects logistic regression models with participant-level and stimulus-level random effects.

Given that our measurement instrument would benefit from validation and pretesting, this study should be understood as an initial empirical exploration, and future work will aim to employ larger, validated item sets and psychometric testing (e.g., reliability analysis, item response theory) to strengthen measurement robustness.

### 6.4 Future work

The future studies should first overcome the limitations of this study which are previously covered. Open research items therefore include the validation of the results of this study. It is advisable for researchers to conduct the same research with different participants (context, culture, gender, age, etc.). In other words, to get clearer picture and avoid the misleading conclusions, the future research should conduct additional surveys covering the rest of population (different age, profession, and education groups) and explore our findings referring to this relationship. Bosnian and Herzegovinian setting provides a specific cultural backdrop but the mechanisms under study are broadly applicable across contexts. Therefore, similar studies should be conducted in other regions and cultures before broader conclusions can be drawn. Furthermore, the future studies should focus on standardization of the cyber hygiene and news veracity judgement questionnaires and afterwards repeat the examination.

Future research should treat cyber hygiene and news veracity detection as distinct constructs and investigate the cognitive, psychological, and contextual factors that influence the news veracity judgement. Studies could examine predictors such as analytical thinking, cognitive reflection, media literacy, prior knowledge, and trust in institutions, as well as developmental and demographic differences across age groups or cultural contexts. Also, incorporating source credibility would provide a more comprehensive understanding of how individuals assess information in realistic contexts. Experimental interventions comparing cyber hygiene training, media literacy training, or combined approaches could clarify which strategies most effectively improve fake news resilience. Longitudinal and real-world studies tracking online behavior would provide insight into how these skills interact over time, while platform specific research could identify contexts where vulnerability is greatest. Overall, future work should move beyond the assumption that technical digital competence automatically protects against fake news and explore integrated approaches that enhance both digital safety and informed judgment.

In addition, future research in this field should move beyond binary true/false classifications and examine broader dimensions of information evaluation, including credibility judgments, bias recognition, uncertainty assessment, and critical engagement with content that is factually mixed or context dependent.

This study can be positioned as an exploratory step that can inform more advanced modeling in subsequent studies. Future research should also consider applying advanced methods to extend the findings and provide a more comprehensive understanding of the factors influencing news veracity judgement.

## Data Availability

The raw data supporting the conclusions of this article will be made available by the authors, without undue reservation.
