# Supplementary material for: Investigation of relation between cyber hygiene and news veracity judgements
Source: Front Public Health. 2026 Oct 26;14:1962217. doi: 10.3389/fpubh.2026.1962217 (PMC13745571; doi:10.3389/fpubh.2026.1962217)
Supplement: Supplementary file 1 [file Supplementary_file_1.docx]

**Supplementary materials**

Table A. The % of correct recognitions of news veracity (each news) in dependence of cyber hygiene behaviour item dealing with devices used for Internet browsing (Heatmap).

|  | News 1 | News 2 | News 3 | News 4 | News 5 | News 6 |
| --- | --- | --- | --- | --- | --- | --- |
| a. Desktop/laptop computer | 63.18 | 46.93 | 50.9 | 28.52 | 77.26 | 82.31 |
| b. Tablet | 64.1 | 48.72 | 53.85 | 20.51 | 69.23 | 84.62 |
| c. Smartphone | 63.27 | 46.26 | 50.68 | 30.95 | 77.55 | 81.29 |
| d. Smart wearables | 74.07 | 62.96 | 59.26 | 25.93 | 74.07 | 81.48 |
| e. Other | 63.16 | 57.89 | 47.37 | 26.32 | 68.42 | 84.21 |

Table B. The % of correct recognitions of news veracity (each news) in dependence of cyber hygiene behaviour item dealing with access to computer/laptop device habits (Heatmap).

|  | News 1 | News 2 | News 3 | News 4 | News 5 | News 6 |
| --- | --- | --- | --- | --- | --- | --- |
| a. Me exclusively | 62.36 | 48.31 | 49.44 | 25.28 | 73.03 | 83.15 |
| b. Me and my parents | 65.96 | 36.17 | 51.06 | 34.04 | 82.98 | 70.21 |
| c. Me and my siblings | 59.46 | 39.19 | 44.59 | 43.24 | 85.14 | 75.68 |
| d. Me and other extended family members | 66.67 | 50 | 50 | 16.67 | 66.67 | 100 |
| e. Me and my partner | 66.67 | 45.83 | 79.17 | 16.67 | 75 | 91.67 |
| f. Me and my friends | 33.33 | 44.44 | 33.33 | 22.22 | 88.89 | 88.89 |

Table C. The % of correct recognitions of news veracity (each news) in dependence of cyber hygiene behaviour item dealing with access to smartphone device habits (Heatmap).

|  | News 1 | News 2 | News 3 | News 4 | News 5 | News 6 |
| --- | --- | --- | --- | --- | --- | --- |
| a. Me exclusively | 61.13 | 43.77 | 49.81 | 31.32 | 79.25 | 81.51 |
| b. Me and my parents | 61.54 | 76.92 | 53.85 | 23.08 | 53.85 | 53.85 |
| c. Me and my siblings | 80 | 60 | 30 | 0 | 80 | 90 |
| d. Me and other extended family members | 0 | 100 | 0 | 100 | 0 | 0 |
| e. Me and my partner | 77.78 | 44.44 | 66.67 | 11.11 | 72.22 | 88.89 |
| f. Me and my friends | 87.5 | 62.5 | 37.5 | 25 | 75 | 87.5 |

Table D. The % of correct recognitions of news veracity (each news) in dependence of cyber hygiene behaviour item dealing application usage habits (Heatmap).

|  | News 1 | News 2 | News 3 | News 4 | News 5 | News 6 |
| --- | --- | --- | --- | --- | --- | --- |
| a. Internet search | 64.77 | 46.62 | 50.18 | 30.25 | 77.58 | 82.21 |
| b. Social networks | 61.84 | 46.64 | 50.18 | 28.62 | 77.39 | 81.63 |
| c. Shopping | 61.88 | 48.07 | 52.49 | 29.28 | 74.59 | 84.53 |
| d. Financial management | 61.45 | 45.78 | 53.61 | 26.51 | 75.9 | 86.75 |
| e. Games | 64.89 | 48.94 | 51.6 | 30.85 | 76.06 | 79.26 |
| f. Security management | 61.4 | 57.89 | 56.14 | 24.56 | 77.19 | 82.46 |
| g. Learning | 63.5 | 43 | 50.5 | 29.5 | 79.5 | 83 |
| h. Other | 67.39 | 56.52 | 60.87 | 21.74 | 69.57 | 84.78 |

Table E. The % of correct recognitions of news veracity (each news) in dependence of cyber hygiene behaviour item dealing with password creating habits (Heatmap).

|  | News 1 | News 2 | News 3 | News 4 | News 5 | News 6 |
| --- | --- | --- | --- | --- | --- | --- |
| a. By combining letters, numbers and symbols and 8 characters | 66.67 | 47.37 | 51.46 | 29.82 | 79.53 | 82.46 |
| b. With at least 8 characters | 60 | 33.33 | 46.67 | 20 | 80 | 93.33 |
| c. From private information  (date of birth, name, etc.) | 53.12 | 43.75 | 34.38 | 40.62 | 78.12 | 75 |
| d. All the above | 58.06 | 40.32 | 53.23 | 25.81 | 77.42 | 77.42 |
| e. None of the above | 61.54 | 57.69 | 57.69 | 34.62 | 61.54 | 80.77 |

Table F. The % of correct recognitions of news veracity (each news) in dependence of cyber hygiene behaviour item dealing with password storing habits (Heatmap).

|  | News 1 | News 2 | News 3 | News 4 | News 5 | News 6 |
| --- | --- | --- | --- | --- | --- | --- |
| a. I store my passwords on a computer or write them down on paper | 64.29 | 37.5 | 49.11 | 31.25 | 82.14 | 80.36 |
| b. I use the same passwords for most occasions | 62.5 | 45.45 | 53.41 | 32.95 | 71.59 | 82.95 |
| c. I store the password on a browser (Mozilla, Chrome, etc.) | 57.78 | 48.89 | 48.89 | 31.11 | 75.56 | 75.56 |
| d. I do not hide my passwords – everyone knows them | 100 | 0 | 0 | 50 | 100 | 50 |
| e. I remember them | 59.74 | 48.05 | 48.05 | 27.27 | 79.22 | 82.47 |
| f. I store my passwords with the help of password manager | 60.92 | 54.02 | 54.02 | 32.18 | 72.41 | 77.01 |

Table G. The % of correct recognitions of news veracity (each news) in dependence of cyber hygiene behaviour item dealing with personal information sharing habits (Heatmap).

|  | News 1 | News 2 | News 3 | News 4 | News 5 | News 6 |
| --- | --- | --- | --- | --- | --- | --- |
| a. Name and surname | 62.77 | 45.74 | 49.65 | 30.14 | 77.66 | 80.5 |
| b. Registration number | 50 | 50 | 50 | 50 | 100 | 66.67 |
| c. Images | 64.04 | 44.74 | 49.12 | 29.39 | 78.95 | 85.09 |
| d. Video | 64.75 | 49.64 | 48.92 | 30.94 | 77.7 | 82.01 |
| e. Documents | 60 | 56 | 62 | 24 | 74 | 88 |
| f. Opinions, feelings,  statements, etc. | 62.38 | 48.51 | 61.39 | 24.75 | 71.29 | 86.14 |
| g. Home address | 51.72 | 55.17 | 58.62 | 24.14 | 79.31 | 86.21 |
| h. Relationship status | 73.17 | 48.78 | 58.54 | 36.59 | 75.61 | 80.49 |
| i. Phone number | 56.57 | 47.47 | 45.45 | 34.34 | 80.81 | 81.82 |
| j. Location | 65.57 | 54.1 | 44.26 | 24.59 | 83.61 | 91.8 |
| k. Bank card number | 61.54 | 53.85 | 53.85 | 7.69 | 69.23 | 100 |
| l. Plans | 78.57 | 57.14 | 57.14 | 28.57 | 71.43 | 85.71 |
| m. Other | 77.78 | 44.44 | 50 | 16.67 | 72.22 | 100 |

Table H. The % of correct recognitions of news veracity (each news) in dependence of cyber hygiene behaviour item dealing with Wi-Fi access habits (Heatmap).

|  | News 1 | News 2 | News 3 | News 4 | News 5 | News 6 |
| --- | --- | --- | --- | --- | --- | --- |
| a. I do not use public Wi-Fi networks | 55.56 | 38.27 | 58.02 | 29.63 | 70.37 | 87.65 |
| b. I use password-protected public Wi-Fi networks | 64.21 | 51.58 | 46.32 | 27.37 | 82.11 | 82.11 |
| c. I use public Wi-Fi networks that are familiar to me | 63.33 | 54.44 | 45.56 | 28.89 | 75.56 | 81.11 |
| d. I use public Wi-Fi networks that are available and free | 68.42 | 47.37 | 48.87 | 32.33 | 83.46 | 76.69 |

Table I. The % of correct recognitions of news veracity (each news) in dependence of cyber hygiene behaviour item dealing with Wi-Fi usage habits (Heatmap).

|  | News 1 | News 2 | News 3 | News 4 | News 5 | News 6 |
| --- | --- | --- | --- | --- | --- | --- |
| a. For social networks (including Viber, WhatsApp, etc.) | 62.89 | 45.36 | 49.83 | 29.21 | 77.32 | 81.44 |
| b. For e-mail communication | 63.22 | 47.13 | 51.72 | 28.74 | 74.71 | 84.48 |
| c. For online shopping | 62.77 | 50.36 | 46.72 | 24.82 | 74.45 | 83.94 |
| d. For banking | 61.32 | 57.55 | 59.43 | 24.53 | 74.53 | 83.96 |
| e. For other application | 65.44 | 52.94 | 50.74 | 30.15 | 77.21 | 80.88 |
